# Inconsistency was more prevalent than reported: an empirical study of 57 networks with multiple treatments using the node-splitting approach and a novel interpretation index

**DOI:** 10.1186/s13643-025-02984-z

**Published:** 2025-11-28

**Authors:** Loukia M. Spineli

**Affiliations:** Midwifery Research and Education Unit, Hannover Medical School, Hannover, Germany

**Keywords:** Inconsistency, Network meta-analysis, Kullback–Leibler divergence, Statistical heterogeneity, Node-splitting approach, Bayesian analysis

## Abstract

**Background:**

Inconsistency has been reported to be ubiquitous in network meta-analysis. However, this evidence is based on statistical tests for inconsistency with well-documented power limitations. A novel interpretation index was developed that is founded on the Kullback–Leibler divergence measure and warrants a semi-objective decision about the extent of inconsistency as acceptably low or material when statistical tests are underpowered. The prevalence of local inconsistency was investigated using the synergy of the Bayesian node-splitting approach with the newly proposed interpretation index. The results were also contrasted with inferences drawn from the ‘standard decision-making approach’ about the presence of inconsistency.

**Methods:**

The *nmadb* R package was considered to obtain the sample of 57 networks on a binary outcome. The Bayesian node-splitting approach was initially applied to each network to estimate the posterior distribution of direct and corresponding indirect effects for each split node alongside the inconsistency factor and between-study standard deviation ($\tau$). Then, the interpretation index was applied to each split node to quantify the average divergence between the direct and indirect effects and determine whether inconsistency was acceptably low or material based on a semi-objectively derived threshold.

**Results:**

The interpretation index revealed material inconsistency in 58% of the split nodes and 81% of the networks compared to the ‘standard decision-making approach’ (whether the 95% credible interval of the inconsistency factor excluded zero inconsistency) that indicated conclusive inconsistency in 4% of the split nodes and 18% of the networks. Material inconsistency was less prevalent for large $\tau$ values and single-study split nodes. Networks with single-study split nodes yielded larger and more imprecise inconsistencies than split nodes with more studies, making inconsistency subject to small-study effects. Such networks were also prone to a spuriously acceptable low inconsistency. Splitting single-study nodes were associated with larger inconsistency with increasing $\tau$ values than splitting nodes with more studies.

**Conclusions:**

Inconsistency should be interpreted cautiously in the presence of single-study comparisons and substantial statistical heterogeneity, as a true inconsistency may be concealed. Local inconsistency should be expected and quantified using a method that aligns with the evidence structure of the network.

**Supplementary Information:**

The online version contains supplementary material available at 10.1186/s13643-025-02984-z.

## Background

Network meta-analysis (NMA), an extension of pairwise meta-analysis for multiple treatments, has undergone fast-paced methodological advances over the last decade, ascribed to the increasing complexity of the evidence base and research questions [1, 2]. Estimating valid indirect effects for treatment comparisons not directly investigated in any study and having consistency across several sources of evidence, forming a connected network of treatments, is integral to the NMA framework and underlies the quality of the generated evidence [3, 4].

Since the introduction of NMA, a rich arsenal of statistical methods of various sophistication has been developed to detect disagreements across the evidence sources, termed *inconsistencies*, which are largely distinguished into local and global methods [5–7]. In brief, local inconsistency methods compare direct with indirect estimates of selected comparisons, forming closed loops of evidence to test the null hypothesis of no inconsistency. Loop-specific and node-splitting approaches are examples of local inconsistency evaluation, with the latter being attractive and more appropriate in large networks with multi-arm studies [5, 8, 9]. Global inconsistency evaluation offers omnibus-like tests to assess inconsistency in the entire network: design-by-treatment inconsistency models, assuming fixed or random inconsistency factors (IFs), and the unrelated mean effects model comprise such methods [5, 6, 10, 11]. Applying both local and global methods has been recommended for offering a comprehensive inconsistency evaluation, especially in networks with complex evidence structures [8, 12].

A common feature of the inconsistency methods is their low power to detect a statistically significant inconsistency [10–13]. Failure to reject the null hypothesis (i.e., a *p*-value above a selected significance threshold or a 95% confidence interval that includes the null value) may invite a misinterpretation of the corresponding results as ‘evidence of absence’, with implications for the quality of the conclusions [14]. Network characteristics, such as the number of included studies, treatments, and closed loops, and the extent of statistical heterogeneity may affect the ability to detect a statistically significant inconsistency [12, 13, 15, 16]. For instance, having sparsely informed closed loops or unrealistically large estimated statistical heterogeneity due to assigning vague prior distributions on the heterogeneity parameter will likely mask an underlying inconsistency, manifesting as a failure to reject the null hypothesis [6, 16].

A novel approach was recently developed to interpret inconsistency as *acceptably low* or *material* based on the results of a *local* evaluation method [17]. The approach is grounded on the Kullback–Leibler divergence (KLD) measure [18] and a semi-objectively derived threshold of acceptably low inconsistency, offering a viable solution to situations where evidence about the presence of inconsistency is inconclusive (i.e., failure to reject the null hypothesis) [17]. In short, the entire distribution of the estimated direct effect for selected comparisons is juxtaposed with the distribution of the corresponding indirect effect to gauge their divergence via the KLD measure, yielding a non-negative scalar index for each selected comparison [17]. Then, each index is compared with the threshold: inconsistency may be acceptably low for the comparison if the corresponding index is below the threshold; otherwise, inconsistency may be material, potentially compromising the validity of results [17].

Several empirical studies have revealed inconsistency to be ubiquitous in published systematic reviews with multiple treatments, affecting 7% to 20% of the investigated networks [8, 12, 19–21]. Due to the low power of the inconsistency methods, we expect inconsistency to be more prevalent than already reported. Therefore, we conducted an empirical study to elucidate the prevalence of local inconsistency using the synergy of the Bayesian node-splitting approach with the newly proposed interpretation index, which warrants a decision about the extent of inconsistency when there is *inconclusive* evidence about inconsistency. The results were also contrasted with the ‘standard decision-making approach,’ where inferences were drawn from assessing whether the 95% credible interval (CrI) of IF (difference between direct and indirect effect) excluded zero inconsistency. Since the interpretation index is a new development in the inconsistency methodology, the present study also elucidated the properties of the index to delineate further advantages and limitations.

## Methods

### Setting up the sample of eligible networks

We used the nmadb R package [22] to build the analysis dataset. The nmadb R package contains a database of 453 networks of multiple treatments on a primary outcome from published systematic reviews with NMA based on the bibliographic study of Petropoulou and colleagues [23]. The database was filtered to obtain the desired sample of networks. Specifically, we opted for networks with (1) arm-level data (i.e., the outcome data are reported for each arm of every study), as we aimed to apply one-stage models, and (2) at least one closed loop not informed by multi-arm studies exclusively. We considered only networks with a binary outcome, the most well-studied in the relevant literature [8, 12, 13, 16, 21]. Logistic regression with binary covariates is prone to separation when the outcome is sparse, leading to infinite results for the regression coefficients [24]. Hence, we excluded studies with event risk below 15% (a cut-off also employed in a relevant simulation study [16]) or above 85% to *eliminate* the implications of possible separation. Of the 453 datasets, 57 (13%) were deemed eligible for analysis. Table 1 illustrates the selection process to obtain the eligible sample of networks.

**Table 1:** The selection process to obtain the 57 eligible networks

Selection process	Counts (%)
Networks found in the *nmadb* [22] database	453
Total excluded for the following reasons	396
Unavailable data^a^	157 (40%)
Investigated continuous, rate and survival outcomes	101 (26%)
Included contrast-based outcome data	10 (3%)
Not analysable after removing studies with questionable data^b^	0 (0%)
All studies had event risk below 15% or above 85%^c^	38 (10%)
Less than three treatments or at least more treatments than studies	51 (13%)
Disconnected networks^d^	6 (2%)
No closed loops or loops informed by multi-arm studies only	33 (8%)
Total eligible for the present study	57 (13%)

^a^No data were extracted for these networks: reading these networks with the function readByID() of the nmadb R package [22] returned NULL

^b^Some networks with binary outcomes contained studies with questionable data that required removal: one network contained studies with zero sample size, and another had studies with non-numeric elements. After removing these studies, the networks were still analysable for having at least four treatments and more studies than treatments

^c^Studies with event risk below 15% or above 85% in at least one treatment arm were also removed to avoid issues with convergence resulting from separation. Some networks contained only such studies and were excluded

^d^Network connectivity was compromised after excluding studies with event risk below 15% or above 85%

### Average Kullback–Leibler divergence for interpreting local inconsistency

Initially, a local inconsistency method was applied to estimate the direct and indirect effects (posterior distributions in the Bayesian framework, means and standard errors in the frequentist framework) for the selected comparisons and calculate their KLD [17]. The split nodes comprised the selected comparisons since we opted for the node-splitting approach. Additional file 1: Methods A [8, 9, 25–28] briefly describes the node-splitting approach introduced by Dias et al. [9] and refined further by van Valkenhoef et al. [25].

The new interpretation approach is based on the conventional assumption that estimated direct and indirect effects follow a normal distribution [6, 17]. Then, the KLD measure for approximating the distribution of the estimated direct effect for a split node $j$ with the distribution of the corresponding indirect effect has the following closed-form expression [18]:

$${D}_{D,I}^{j}=\frac{1}{2}\left[\frac{{\widehat{s}}_{D}^{2}}{{\widehat{s}}_{I}^{2}}+\frac{{\left({\widehat{\mu }}_{D}-{\widehat{\mu }}_{I}\right)}^{2}}{{\widehat{s}}_{I}^{2}}-1+ln\left(\frac{{\widehat{s}}_{I}^{2}}{{\widehat{s}}_{D}^{2}}\right)\right]$$

with ${\widehat{\mu }}_{D}$ and ${\widehat{\mu }}_{I}$ referring to the posterior mean (or mean estimate in the frequentist framework) of the direct and indirect effect, respectively, and ${\widehat{s}}_{D}^{2}$ and ${\widehat{s}}_{I}^{2}$ being the corresponding posterior variances. The difference ${\widehat{\mu }}_{D}-{\widehat{\mu }}_{I}$ quantifies the inconsistency for the investigated split node. The index $j$ is dropped from the parameters above to ease presentation. Subsequently, ${D}_{I,D}^{j}$ is the KLD for approximating the distribution of the indirect effect for a split node $j$ with the distribution of the corresponding direct effect, defined as follows:

$${D}_{I,D}^{j}=\frac{1}{2}\left[\frac{{\widehat{s}}_{I}^{2}}{{\widehat{s}}_{D}^{2}}+\frac{{\left({\widehat{\mu }}_{D}-{\widehat{\mu }}_{I}\right)}^{2}}{{\widehat{s}}_{D}^{2}}-1+ln\left(\frac{{\widehat{s}}_{D}^{2}}{{\widehat{s}}_{I}^{2}}\right)\right]$$

The average of ${D}_{D,I}^{j}$ and ${D}_{I,D}^{j}$ yields the interpretation index, ${D}^{j}$, that captures the average divergence between the distribution of the direct and indirect effects for the split node $j$. ${D}^{j}$ is non-negative, with values closer to 0 indicating a smaller divergence between the compared evidence sources and low inconsistency. The index ${D}^{j}$ and inconsistency have a parabola-like association, ${D}^{j}=a{x}^{2}+bx+c$, with $x={\widehat{\mu }}_{D}-{\widehat{\mu }}_{I}$ and $b=0$ (Additional file 1: Methods B).

#### Threshold of acceptably low inconsistency

Since KLD has no ‘natural’ cut-off value that indicates a small divergence, we devised a threshold for ${D}^{j}$ to signify acceptably low inconsistency by leveraging the *opinion elicitation* framework of Spiegelhalter and colleagues [17, 29]. Specifically, assuming the estimated direct and indirect effects of a split node follow a normal distribution with the same mean but variance equal to ${\tau }^{2}$ and ${2\tau }^{2}$, respectively, their difference would follow a normal distribution with zero mean (consistency on average) and variance ${3\tau }^{2}$ [17]. Then, their absolute difference above zero would follow a half-normal distribution with a scale parameter $\sqrt{3}\tau$ [17, 29]. The median of this half-normal distribution is ${\Phi }^{-1}\left(0.75\right)\times \sqrt{3}\tau \cong 1.17\tau$ [17]. Replacing ${\widehat{\mu }}_{D}-{\widehat{\mu }}_{I}$ with $1.17\tau$, ${\widehat{s}}_{D}^{2}$ with ${\tau }^{2}$ and ${\widehat{s}}_{I}^{2}$ with ${2\tau }^{2}$ in the equations for ${D}_{D,I}^{j}$ and ${D}_{I,D}^{j}$ and calculating their average yields ${D}^{j}$ equal to approximately 0.64, which comprises the threshold of acceptably low inconsistency [17]. Additional file 2: Fig. S1 illustrates two scenarios where the average IF is virtually zero; however, the extent of overlap of the probability distributions for the direct and indirect effects differs, yielding different conclusions about inconsistency. In the discussion, we express our views on the routine use of fixed thresholds and provide guidance on the best application of the newly proposed index when inferring concerns about inconsistency.

### Model implementation

The node-splitting approach and index ${D}^{j}$ were employed using the rnmamod R package [28] (R software version 4.5.1 [30]). Three chains were considered with 20,000 iterations, 2000 burn-in, and thinning at 10 to conduct the node-splitting approach. Convergence was evaluated for each network by summarising the Gelman-Rubin convergence diagnostic ($\widehat{R}$) [31] for the monitored parameters (direct and indirect effects, IF, and between-study standard deviation) across the split nodes: maximum $\widehat{R}$ below 1.1 indicated convergence in the corresponding network.

Binary outcomes were analysed in the log odds ratio (OR) scale using empirical prior distributions for the between-study variance, ${\tau }^{2}$, tailored to the investigated outcome and treatment-comparison type (Additional file 1: Methods C) [22, 32]. Following the critical views of Bakbergenuly et al. [33] on the current meta-analysis models for risk ratio and the lack of empirical priors for ${\tau }^{2}$ under risk ratio and risk difference, we did not investigate the impact of other effect measures for binary outcomes on detecting inconsistency.

### Presentation of the results

The characteristics of the networks were summarised in a table using the median and range for the quantitative and the absolute and relative frequencies for the qualitative characteristics. For each network and split node, we considered the ${D}^{j}$ value, and the summaries of the posterior distribution of IF, direct and indirect effects, and $\tau$. Box plots with integrated dots were created to summarise the posterior distribution of IF and (in)direct estimates. Scatter plots were drawn to investigate the association of ${D}^{j}$ with the posterior results of the IF and $\tau$. The association between ${D}^{j}$ and the KLD to approximate the distribution of either effect (i.e., ${D}_{D,I}^{j}$ and ${D}_{I,D}^{j}$) was also investigated using scatter plots. The extent of $\tau$ in the log OR scale was considered using the suggested cut-offs [29]: low ($\tau \le 0.1$), reasonable ($0.1<\tau <0.5$), fairly high ($0.5\le \tau \le 1.0$), and fairly extreme ($\tau>1.0$).

The conclusions about inconsistency were drawn for each split node by (1) comparing the corresponding ${D}^{j}$ value with 0.64 (acceptably low or material inconsistency) and (2) assessing whether the 95% CrI of the corresponding IF excluded or included zero inconsistency (conclusive or inconclusive inconsistency). Conclusive inconsistency corresponded to a 95% CrI of IF that excluded zero, implying present inconsistency; otherwise, inconsistency was inconclusive.

We devised the following rule to judge a network as potentially inconsistent: at least one split node should have ${D}^{j}\ge 0.64$ or a 95% CrI of IF that excludes zero. Only ${D}^{j}$ can indicate the consistent networks (if any): all split nodes should have ${D}^{j}<0.64$. When all split nodes were associated with a 95% CrI of IF that included zero, inconsistency was considered *inconclusive* for the corresponding network. Using stacked bar plots, we illustrated the percentage of networks and split nodes with acceptably low, material, conclusive, and inconclusive inconsistency. All graphs were created using the ggplot2 and ggpubr R packages [34, 35].

## Results

Table 2 summarises the characteristics of the analysed networks. The studies ranged from 6 to 104 (median 16), with a median sample size of 153 participants (range 10–9331) and a median of 7 treatments (range 4–28). Multi-arm studies populated 77% of the networks, ranging from 1 to 30% (median: 11%) of their studies. Almost all networks ($n=56$) included at least one single-study comparison. The number of split nodes ranged from 1 to 40 (median 3), with 16 networks (28%) containing only one split node. Most networks ($n=49$; 86%) included at least one single-study split node, with 14 networks having only single-study split nodes. Two in three networks investigated a beneficial outcome. Semi-objective primary outcomes ($n=24$; 42%) and comparisons of pharmacological treatments with placebo ($n=39$; 68%) governed the analysed networks.

**Table 2:** Characteristics of the 57 eligible networks

Characteristic	Summary^a^
Number of studies	16 (6, 104)
Two-arm studies (%)	94 (70, 100)
Multi-arm studies (%)	11 (1, 30)
Study sample size	153 (10, 9331)
Number of treatments	7 (4, 28)
Observed comparisons^b^ (%)	47 (10, 100)
Single-study comparisons^c^ (%)	50 (8, 100)
Number of split nodes	3 (1, 40)
Single-study split nodes (%)	62 (8, 100)
Outcome was beneficial	
Yes (%)	38 (67)
No (%)	19 (33)
Outcome type	
Objective (%)	19 (33)
Semi-objective (%)	24 (42)
Subjective (%)	14 (25)
Treatment-comparison type	
Pharmacological *vs*. placebo	39 (68)
Pharmacological *vs*. pharmacological	11 (19)
Non-pharmacological *vs*. any	7 (12)

^a^Quantitative characteristics were summarised using the median and range in parenthesis (minimum and maximum). Qualitative characteristics were summarised using absolute frequencies and relative frequencies in parenthesis

^b^There was one fully connected network; after excluding this network, the percentage median remained at 47 (% range 10–90)

^c^One network contained only single-study comparisons; after excluding this network, the percentage median remained at 50 (% range 8–80)

### Convergence and distribution of monitored parameters

**Fig. 1:**
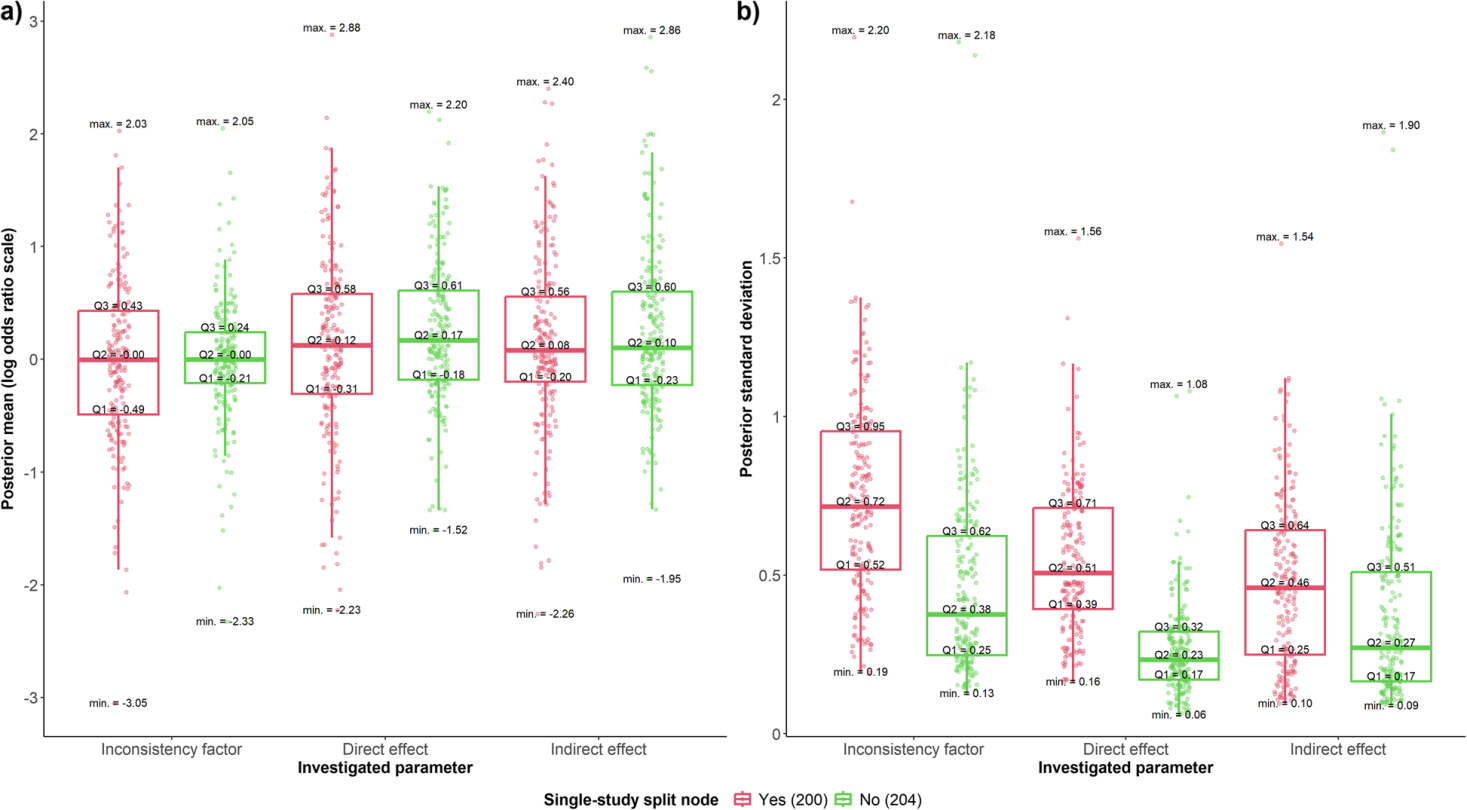
Box plots with integrated dots for the **a** posterior mean and **b** posterior standard deviation of inconsistency factor, direct and indirect effects in the log odds ratio scale based on 404 split nodes in 57 networks. Red and green refer to 200 single-study split nodes and 204 split nodes with more studies

Convergence was achieved in all networks for all split nodes. Figure 1 presents the posterior distribution of the (in)direct effects and IF. Single-study split nodes had larger posterior standard deviations than split nodes with more studies (Fig. 1b); the latter had smaller inconsistencies and slightly larger indirect effects than single-study split nodes (Fig. 1a). Additional file 2: Fig. S2 illustrates the association between the parameters’ posterior standard deviations and means or medians, accounting for the split node size.

### Prevalence of inconsistency based on 95% CrI of IF and index ${D}^{j}$

**Fig. 2:**
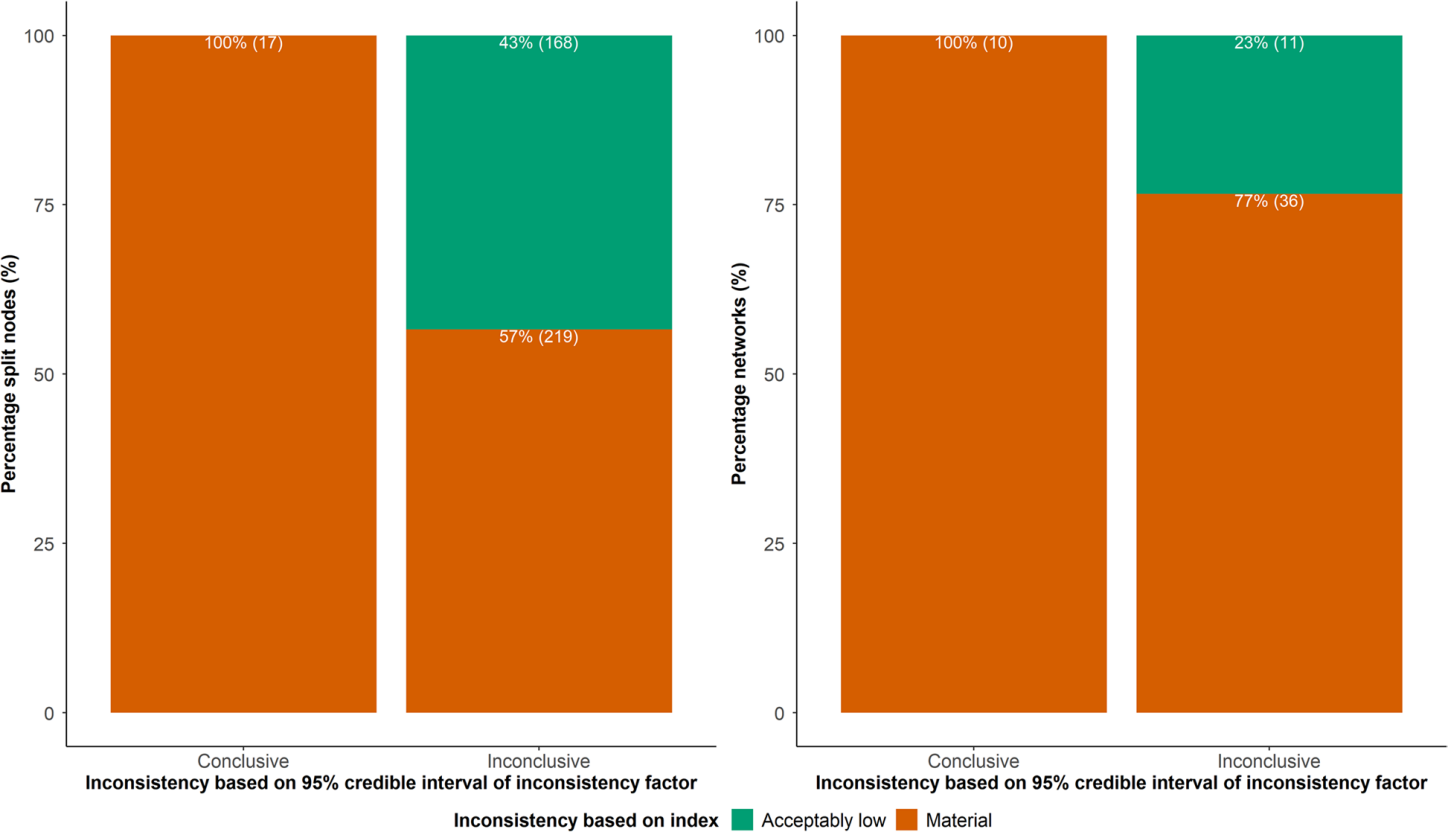
Stacked bar plots with the conclusions about inconsistency based on the interpretation index ${D}^{j}$ and the 95% credible interval of the inconsistency factor in 57 networks with 404 split nodes. Percentages refer to split nodes and networks with acceptably low (${D}^{j}<0.64$) or material inconsistency based on the index ${D}^{j}$ out of those with conclusive (95% credible interval excludes zero inconsistency) and inconclusive inconsistency

Almost all split nodes (96%; 387 out of 404) and most networks (82%; $n=47$) were associated with inconclusive inconsistency for having a 95% CrI of IF that included zero inconsistency (Fig. 2). Fifty-seven percent ($n=219$) of the ‘inconclusive’ nodes and 77% ($n=36$) of the ‘inconclusive’ networks were associated with a material inconsistency. All 17 split nodes and 10 networks with conclusive inconsistency had material inconsistency. Overall, 81% ($n=46$) of the networks and 58% ($n=236$) of the split nodes had material inconsistency compared to 18% ($n=10$) of the networks and 4% ($n=17$) of the split nodes with conclusive inconsistency. Split nodes with more studies were associated with slightly more conclusive nodes and slightly more nodes with material inconsistency than single-study split nodes (Additional file 2: Fig. S3).

### Association of the index ${D}^{j}$ with the posterior distribution of inconsistency

**Fig. 3:**
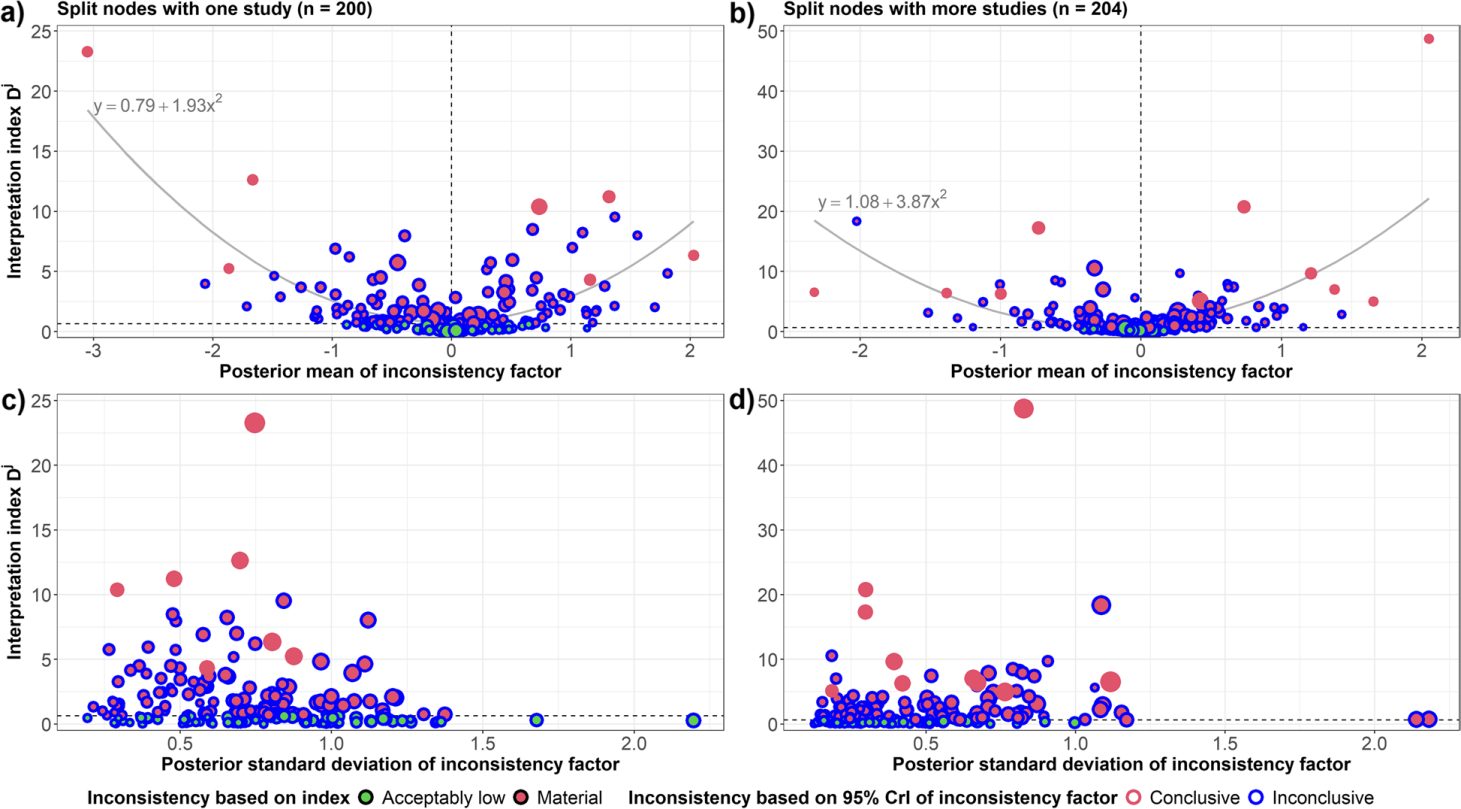
Scatter plots of the interpretation index ${D}^{j}$ against the posterior mean and standard deviation of the inconsistency factor in 200 single-study split nodes (plots **a** and **c**) and 204 split nodes with more studies (plots **b **and **d**). Each dot is a split node, with the dot size proportional to the inverse of the posterior variance (plots **a **and **b**) and the posterior mean of inconsistency factor (plots **c **and **d**). Red dots with red frames correspond to material (${D}^{j}\ge 0.64$) and conclusive (95% credible interval excludes zero inconsistency) inconsistency, red or green dots with blue frames refer to material or acceptably low but inconclusive inconsistency

Figure 3 presents the association of ${D}^{j}$ with the posterior mean and standard deviation of IF for split nodes with one or more studies. The parabola-like association between ${D}^{j}$ and IF was twice wider upwards in split nodes with more studies, attributed to the outlying split node (Fig. 3b). Except for the outlying split node (of 17 studies in a network with the remaining comparisons including one or two studies), the ${D}^{j}$ values were similarly distributed in both split node groups, especially their midspread (Additional file 2: Fig. S4). ${D}^{j}$ values over five were outliers in both split node groups, with ${D}^{j}>15$ deviating substantially from the rest (Additional file 2: Fig. S4).

Overall, split nodes with large IF values (in either direction) and smaller posterior standard deviations were more likely to yield conclusive inconsistency (95% CrI of IF excluded zero inconsistency); otherwise, inconsistency was likely to be inconclusive (Fig. 3). This pattern was less obvious in split nodes with material inconsistency; however, these split nodes were more likely to be scattered below and above a zero IF in a wide range of posterior standard deviations of IF, creating an albatross-like plot (Additional file 2: Fig. S5a): material inconsistency in split nodes with more studies was relatively smaller (IQR of IF − 0.39–0.43) than in single-study split nodes (IQR of IF − 0.66–0.65).

Among the split nodes with ${D}^{j}<0.64$, those with more studies were distributed in a narrower range of IF values (range − 0.41–0.24) than single-study split nodes (range − 0.88–1.13) (Fig. 3a, b). However, both split node groups were associated with a wide range of posterior standard deviations of IF (Fig. 3c, d). Split nodes with smaller posterior standard deviations of IF were plotted near zero inconsistency, and those with larger posterior standard deviations of IF were scattered below and above zero inconsistency, creating a funnel-like plot (Additional file 2: Fig. S5b): that pattern was more evident in single-study split nodes for having a wider range of posterior means and standard deviations for IF.

### Association of index ${D}^{j}$ with the KLD of approximating the (in)direct effects

**Fig. 4:**
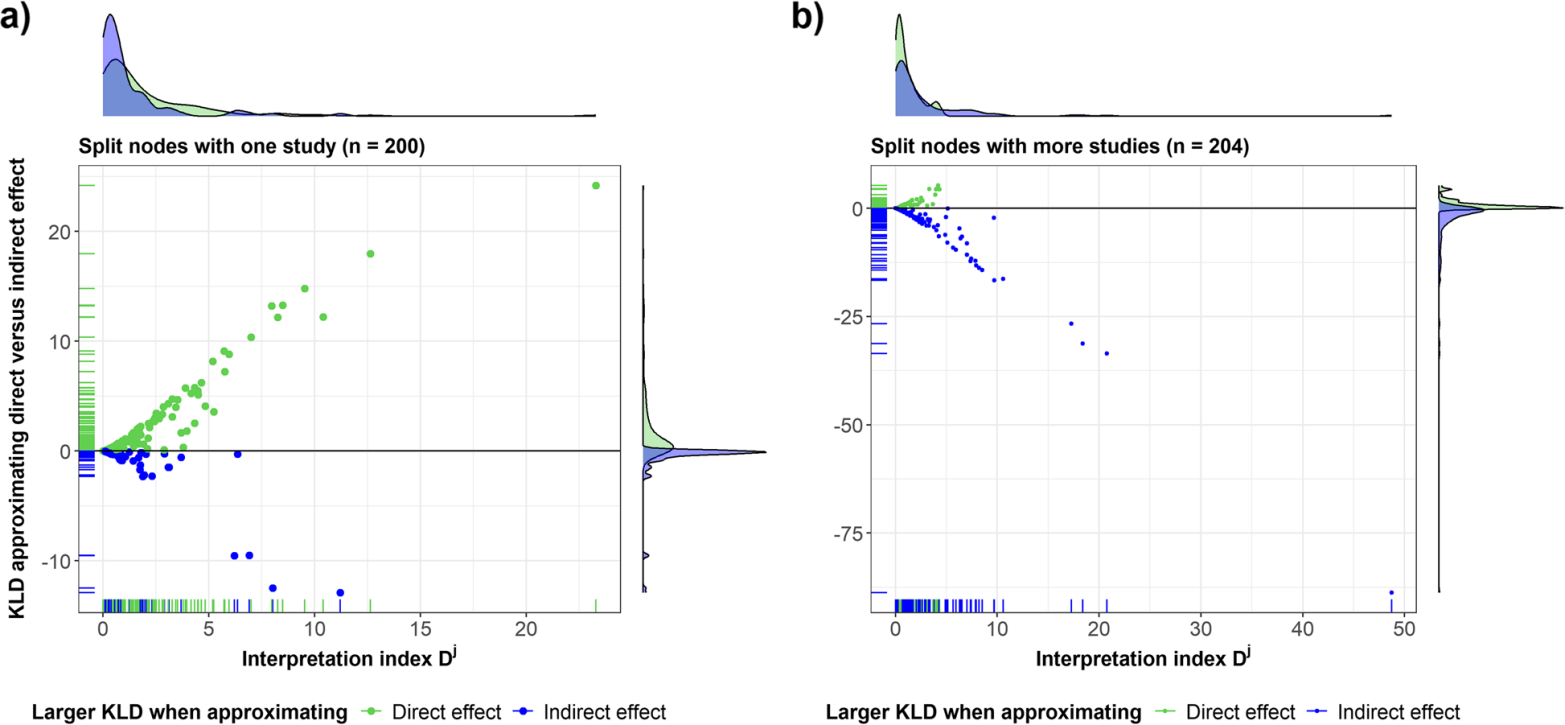
Scatter plot of the difference between the Kullback–Leibler divergence of approximating the direct effects (${D}_{D,I}^{j}$) and Kullback–Leibler divergence of approximating the indirect effects (${D}_{I,D}^{j}$) against the interpretation index ${D}^{j}$ in **a** 200 single-study split nodes and **b** 204 split nodes with more studies. Both scatter plots have a black horizontal line at zero difference. A density plot of the distribution of ${D}_{D,I}^{j}>$ ${D}_{I,D}^{j}$ (green area) and ${D}_{D,I}^{j}<$ ${D}_{I,D}^{j}$ (blue area) is appended on the right of the scatter plots, and a density plot of the distribution of ${D}^{j}$ for ${D}_{D,I}^{j}>$ ${D}_{I,D}^{j}$ (green area) and ${D}_{D,I}^{j}<$ ${D}_{I,D}^{j}$ (blue area) is appended above the scatter plots

Figure 4 illustrates the association of index ${D}^{j}$ with the difference between ${D}_{D,I}^{j}$ and ${D}_{I,D}^{j}$ for split nodes with single and many studies. The size of the split nodes appeared to have implicated ${D}_{D,I}^{j}$ and ${D}_{I,D}^{j}$ differently. Specifically, across single-study split nodes (Fig. 4a), ${D}_{I,D}^{j}$ had overall smaller values than ${D}_{D,I}^{j}$ (blue dots) and, hence, contributed less to ${D}^{j}$. Since single-study split nodes had more imprecise direct effects than the corresponding indirect effects, approximating direct with indirect effects resulted in larger ${D}_{D,I}^{j}$ values compared to ${D}_{I,D}^{j}$.

For split nodes with more studies (Fig. 4b), ${D}_{I,D}^{j}$ had larger values than ${D}_{D,I}^{j}$, and thus, ${D}_{I,D}^{j}$ contributed more to ${D}^{j}$. Indirect effects of split nodes with more studies were more imprecise than the direct effects (Fig. 1b); hence, approximating indirect effects with the corresponding direct effects yielded larger ${D}_{I,D}^{j}$ values. This differential impact of the split node size on ${D}_{D,I}^{j}$ and ${D}_{I,D}^{j}$ was almost negligible after restricting to split nodes with ${D}^{j}<0.64$, with ${D}_{D,I}^{j}-{D}_{I,D}^{j}$ ranging from − 0.55 to 0.55, owing to the low threshold (Additional file 2: Fig. S6).

### Association of the index ${D}^{j}$ with the posterior median of $\tau$

**Fig. 5:**
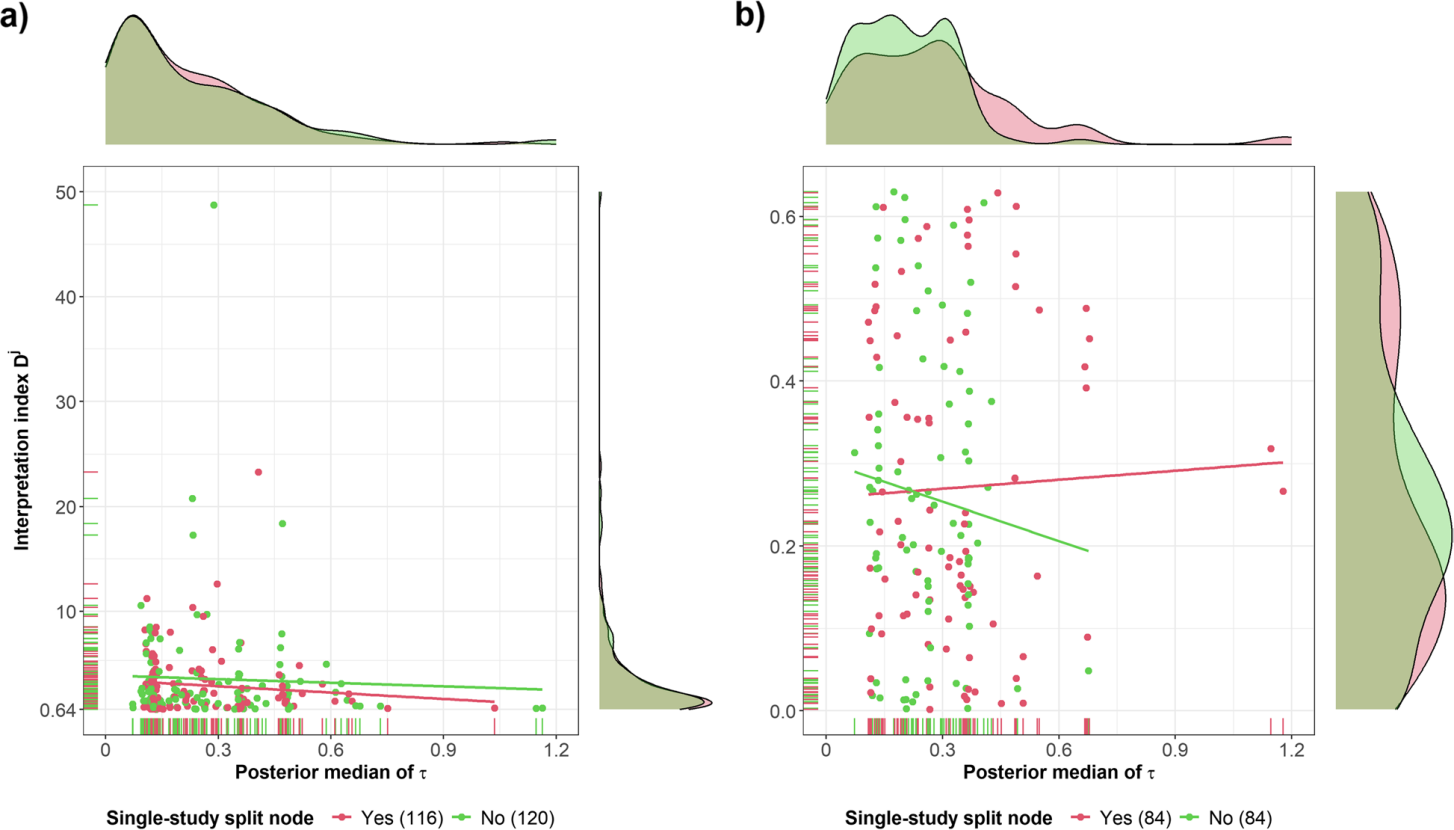
Scatter plot of the interpretation index ${D}^{j}$ against the posterior median of $\tau$ for split nodes with **a** material inconsistency (${D}^{j}\ge 0.64$), and **b** acceptably low inconsistency. Red and green refer to 200 split nodes with one study and 204 split nodes with more studies. A density plot of the distribution of $\tau$ for each split node group is appended above the scatter plots, and a density plot of the distribution of ${D}^{j}$ for each split node group is appended on the right of the scatter plots

In both split node groups, almost all split nodes were associated with reasonable statistical heterogeneity ($0.1<\tau <0.5$), and there was no direct association between the index ${D}^{j}$ and $\tau$ (Fig. 5). Especially for material inconsistency, the distributions of ${D}^{j}$ and posterior median of $\tau$ almost overlapped for both split node groups (Fig. 5a). Overall, among the split nodes with ${D}^{j}<0.64$ (Fig. 5b), those with more studies had a lower $\tau$ (range 0.07–0.68, median 0.26) than single-study split nodes (range 0.11–1.18, median 0.32).

Interestingly, 12 split nodes with fairly high or extreme statistical heterogeneity ($\tau \ge 0.5$) were associated with acceptably low inconsistency (Fig. 5b). For most split nodes, the range of direct and indirect effects covered large log OR values, leading to large or imprecise inconsistencies (Additional file 2: Fig. S7). Apart from the split nodes with ${D}^{j}<0.16$ for having sufficiently overlapping probability densities, in the remaining split nodes, hypothetically smaller $\tau$ values may have led to larger information loss and ${D}^{j}$ values beyond 0.64. Consequently, the large $\tau$ values may have concealed an underlying inconsistency.

Additional file 2: Fig. S8 illustrates the positive association between IF and $\tau$ for split nodes with material (plot a) and acceptably low inconsistency (plot b). Among the split nodes with ${D}^{j}\ge 0.64$, inconsistency increased at a similar rate in both split node groups (Additional file 2: Fig. S8a). On the contrary, among the split nodes with ${D}^{j}<0.64$, the incremental rate of inconsistency was steeper for single-study split nodes than for split nodes with more studies, because they had a larger range of IF and $\tau$ values (Additional file 2: Fig. S8b).

## Discussion

Inconsistency should be expected and quantified using a local method that aligns with the evidence structure complexity of the investigated network. We preferred the node-splitting approach for properly addressing the multi-arm studies [9], which were prevalent in our sample of networks. Then, by calculating the index ${D}^{j}$ for each split node and comparing it with the threshold of acceptably low inconsistency, we found material inconsistency to dominate the networks and split nodes. On the contrary, the ‘standard decision-making approach’ about inconsistency based on the 95% CrI of IF indicated only 10 networks and a handful of split nodes with conclusive inconsistency, with inconsistency being inconclusive in the remaining networks and split nodes.

Using the index ${D}^{j}$, the present study revealed that inconsistency was more prevalent than reported in the literature, which was primarily based on statistical testing in the frequentist framework. The reportedly acknowledged power limitations of testing for inconsistency were evident in the relevant literature. For instance, Song et al. [19] identified conclusive inconsistency ($\text{p-value} < 0.05$) in *only* 7% of the triangles employing the loop-specific approach. In a larger subsequent study, the authors found conclusive inconsistency in 14% of the triangles [21]. Veroniki et al. [8] considered 40 networks with at least four treatments and detected a conclusive inconsistency in *only* 2% to 9% of the loops using the loop-specific approach and 13% of the networks using the design-by-treatment inconsistency model. In the largest empirical study on inconsistency assessment, the authors found conclusive inconsistency in *only* 14% and 20% of the 201 networks at 0.05 and 0.10 significance levels [12].

Being a function of IF, the index ${D}^{j}$ shared the same features as the ‘standard decision-making approach’ about inconsistency: material inconsistency was less prevalent for large $\tau$ values and in single-study split nodes, despite having a larger IF than split nodes with more studies. The negative association between $\tau$ and detecting inconsistency is well-documented in the relevant literature [13, 15, 16, 36], as well as the difficulty in detecting inconsistency in loops with one or more single-study comparisons [12, 13, 16]. In an early small-scale empirical study, Veroniki et al. [8] found that, under a within-loop heterogeneity, loops with a single-study comparison (called ‘typical loops’) were more likely to signal inconsistency than loops with larger comparisons; however, the inconsistency rate decreased in the typical loops under a within-network heterogeneity, aligning with our findings, where we assumed a common $\tau$ across comparisons. In their empirical study, Song et al. [21] found statistically significant inconsistency to be associated with loops informed by few studies, contradicting our findings and those of other studies [12, 13, 16]. Since the authors used the DerSimonian and Laird heterogeneity estimator, which tends to underestimate $\tau$ in small meta-analyses, their result is likely an artefact of the selected heterogeneity estimator. The authors also acknowledged the potential of small-study effect confounding with these findings [21].

Restricting to split nodes with ${D}^{j}<0.64$ gave further insights: single-study split nodes yielded larger and more imprecise IF than split nodes with more studies, making inconsistency prone to small-study effects. Furthermore, splitting single-study nodes led to an increased IF at a higher rate with increasing $\tau$ than splitting nodes with more studies. These likely false negative findings coincide with the low power of statistical tests for inconsistency reported in Song et al. [13] and Veroniki et al. [8] for scenarios that include a ‘typical loop’ and substantial $\tau$. The recent simulation study by Qin et al. [37] suggested that the low power of the inconsistency test (based on the side-splitting approach) may stem from model misspecification, which may lead to mistaken inconsistency for statistical heterogeneity. Consequently, interpreting inconsistency in the presence of single-study split nodes and substantial $\tau$ requires great caution because a true inconsistency may be concealed based on the index ${D}^{j}$ or the ‘standard decision-making approach’.

For each split node, Dias et al. [9] reported the posterior distribution of the IF with the two-sided Bayesian *p*-value and the posterior distribution of direct and indirect estimates, τ, and goodness of fit parameters. However, in published systematic reviews with NMA, conclusions about inconsistency are *primarily* based on assessing the 95% CrI of IF, which we termed the ‘standard decision-making approach’. Therefore, we did not consider the Bayesian *p*-value for IF (also known as conflict $P$ between the direct and indirect evidence [9]) in the present empirical study. Since the Bayesian *p*-value does not have the same interpretation as the frequentist *p*-value, comparing it with significance thresholds, such as 5% or 10%, would be inappropriate.

We excluded studies with event risks that may invite convergence issues to obtain posterior distributions of the log ORs that covered ‘plausible’ values. However, the variability in the baseline risks across the studies and the prevalence of single-study comparisons led to some estimated direct and indirect effects being warily imprecise, implicating the ability to detect conclusive inconsistency and leading to spuriously small ${D}^{j}$ values. Since network sparsity is common in medical research [12], imprecision in the estimated effects would be inevitable, requiring a different route for interpreting results from inconsistency evaluation. For instance, determining a range of clinically important effects for the investigated outcome when presenting the probability densities of the (in)direct effects for split nodes would aid in deciding whether judgments about the inconsistency extent (being acceptably low or material) are sensible, similar to evaluating imprecision using the CINeMA approach [38].

We used a threshold of 0.64 for all analysed networks to interpret the index results as acceptably low or material inconsistency. While the specification of the threshold relies on statistical grounds [17, 29], it may be misleading when applied as a blanket rule across networks with varying complexity, data sparsity, and statistical heterogeneity. Although appealing for facilitating binary decisions, selecting a fixed numerical cut-off is challenging (besides being potentially misleading), as a larger threshold will likely result in more split nodes with acceptably low inconsistency, while a smaller threshold will exacerbate material inconsistency. Instead, ranges of values, often overlapping, should be preferred to ensure applicability across diverse research questions. Following from our aforementioned suggestion, one could specify a range of clinically acceptable inconsistencies to replace ${\widehat{\mu }}_{D}-{\widehat{\mu }}_{I}$ in ${D}_{D,I}^{j}$ and ${D}_{I,D}^{j}$ and calculate ${D}^{j}$ assuming ${\widehat{s}}_{D}^{2}={\tau }^{2}$ and ${\widehat{s}}_{I}^{2}=2{\tau }^{2}$, with $\tau$ based, for example, on the cut-offs of Spiegelhalter and colleagues [29]. This will lead to several ranges of ${D}^{j}$ depending on the different $\tau$ values, therefore, promoting a sensitivity analysis. Since the index also accounts for the uncertainty in the estimates, it is a more appropriate measure than using only the range of clinically acceptable inconsistencies to judge concerns about inconsistency.

The present study has strengths and limitations. We compared the interpretation index with a semi-objective threshold of acceptably low inconsistency. However, in sparse networks with large $\tau ,$ the threshold of 0.64 may not be a sensible choice for increasing the likelihood of misjudging a split node as having acceptably low inconsistency. Furthermore, we employed a somewhat arbitrary rule to classify the networks as having either acceptably low or material inconsistency. Although this rule facilitated gauging the prevalence of inconsistency in our sample of networks, it has no practical value and may also oversimplify the interpretation of inconsistency, as evidence of potential inconsistency should prompt researchers to explore the possible sources [12, 15]. The sample of networks was carefully selected to mitigate separation effects in the estimated parameters resulting from studies with event risks below 15% or above 85%. However, this decision led to a relatively smaller sample of analysed networks. The index ${D}^{j}$ is the product of the posterior distributions of direct and indirect effects; therefore, any implications of the event frequency on the performance of IF have direct repercussions on the proposed index. Inconsistency measures are known to be the most unreliable in networks containing studies with rare events. Veroniki et al. [16] found that the performance of the inconsistency test deteriorates with rare events, challenging inconsistency detection. Therefore, determining clinically acceptable inconsistencies to translate them into index thresholds is crucial in networks with rare event studies to aid in judging any inconsistency concerns and also avoid misinterpreting inevitable false negative results as evidence of consistency. Lastly, other outcome types were less prevalent in the nmadb database, and thus, they were not considered. We do not expect to find different patterns in the results for other outcome types; however, whether the inconsistency rate would be as high as for binary outcomes is unclear.

## Conclusions

Local inconsistency was prevalent in our sample of networks, affecting more networks than revealed by the relevant literature. The newly proposed interpretation index for local inconsistency aids in determining whether the inconsistency is acceptably low or material when the ‘standard decision-making approach’ provides inconclusive evidence about inconsistency. However, similar to the ‘standard decision-making approach’, the index can fail to detect a material inconsistency when substantial statistical heterogeneity or single-study comparisons govern the network, as the estimated (in)direct effects and IF would be imprecise. Analysts should be careful when investigating networks with sparsely informed comparisons or substantial variation in the event rate, as these networks might yield imprecise (and nonsensical) treatment effects, causing a misleading inconsistency evaluation. Illustrating the probability densities of the direct and indirect effects for the split nodes can unveil whether a material inconsistency is likely to be concealed.

## Supplementary Information

Additional file 1: Methods A. Local inconsistency evaluation using the node-splitting approach. Methods B. Parabola-like association of the index ${D}^{j}$ with inconsistency. Method C. Selecting predictive distributions for the between-study variance.

Additional file 2: Figure S1. An illustrative example of the interpretation index ${D}^{j}$ under an acceptably low and material inconsistency. Figure S2. Scatter plots of the posterior standard deviation against the estimated a) direct effects, b) indirect effects, c) inconsistency factor, and d) between-study standard deviation of 404 split nodes in 57 networks. Figure S3. Stacked bar plots on the conclusions about inconsistency based on the interpretation index ${D}^{j}$ and the 95% credible interval of inconsistency factor. Figure S4. Violin plots on the distribution of the interpretation index ${D}^{j}$ among split nodes with one and more studies. Figure S5. Scatter plot of the posterior standard deviation of inconsistency against the posterior mean of inconsistency factor for split nodes with a) material inconsistency, and b) acceptably low inconsistency. Figure S6. Scatter plot of the interpretation index ${D}^{j}$ against ${D}_{D,I}^{j}$ (green dots) and ${D}_{I,D}^{j}$ (blue dots) in split nodes with a) one study and b) more studies with acceptably low inconsistency. Figure S7. Posterior densities of the direct and indirect log odds ratios for 12 split nodes with ${D}^{j}<0.64$ and fairly high or extreme statistical heterogeneity. Figure S8. Scatter plot of the posterior mean of inconsistency factor against the posterior median of $\tau$ for split nodes with a) material inconsistency, and b) acceptably low inconsistency.

## Data Availability

The data supporting the present study’s findings and the functions to create the tables and figures of the present study are publicly available at https://github.com/LoukiaSpin/Local-inconsistency-prevalence-Kullback-Leibler-measure.git.

## Abbreviations

IQR: Interquartile range
KLD: Kullback-Leibler divergence
NMA: Network meta-analysis
OR: Odds ratio

## References

[1] Shi J, Gao Y, Ming L, Yang K, Sun Y, Chen J, et al. A bibliometric analysis of global research output on network meta-analysis. BMC Med Inform Decis Mak. 2021;21(1):144. 33941172 10.1186/s12911-021-01470-5 PMC8094555

[2] Ades AE, Welton NJ, Dias S, Phillippo DM, Caldwell DM. Twenty years of network meta-analysis: continuing controversies and recent developments. Res Synth Methods. 2024;15(5):702–27. 38234221 10.1002/jrsm.1700

[3] Caldwell DM, Ades AE, Higgins JP. Simultaneous comparison of multiple treatments: combining direct and indirect evidence. BMJ. 2005;331(7521):897–900. 16223826 10.1136/bmj.331.7521.897 PMC1255806

[4] Jansen JP, Naci H. Is network meta-analysis as valid as standard pairwise meta-analysis? It all depends on the distribution of effect modifiers. BMC Med. 2013;11:159. 23826681 10.1186/1741-7015-11-159 PMC3707819

[5] Efthimiou O, Debray TP, van Valkenhoef G, Trelle S, Panayidou K, Moons KG, et al. GetReal in network meta-analysis: a review of the methodology. Res Synth Methods. 2016;7(3):236–63. 26754852 10.1002/jrsm.1195

[6] Dias S, Welton NJ, Sutton AJ, Caldwell DM, Lu G, Ades AE. Evidence synthesis for decision making 4: inconsistency in networks of evidence based on randomized controlled trials. Med Decis Making. 2013;33(5):641–56. 23804508 10.1177/0272989X12455847 PMC3704208

[7] Nikolakopoulou A, White IR, Salanti G. Chapter 10: Network meta-analysis. In: Schmid CH, Stijnen T, White IR, editors. *Handbook of Meta-analysis*. 1st ed. Chapman and Hall/CRC; 2020. p. 187–217.

[8] Veroniki AA, Vasiliadis HS, Higgins JP, Salanti G. Evaluation of inconsistency in networks of interventions. Int J Epidemiol. 2013;42(1):332–45. 23508418 10.1093/ije/dys222 PMC5411010

[9] Dias S, Welton NJ, Caldwell DM, Ades AE. Checking consistency in mixed treatment comparison meta-analysis. Stat Med. 2010;29(7–8):932–44. 20213715 10.1002/sim.3767

[10] Higgins JP, Jackson D, Barrett JK, Lu G, Ades AE, White IR. Consistency and inconsistency in network meta-analysis: concepts and models for multi-arm studies. Res Synth Methods. 2012;3(2):98–110. 26062084 10.1002/jrsm.1044 PMC4433772

[11] Jackson D, Barrett JK, Rice S, White IR, Higgins JP. A design-by-treatment interaction model for network meta-analysis with random inconsistency effects. Stat Med. 2014;33(21):3639–54. 24777711 10.1002/sim.6188 PMC4285290

[12] Veroniki AA, Tsokani S, White IR, Schwarzer G, Rücker G, Mavridis D, et al. Prevalence of evidence of inconsistency and its association with network structural characteristics in 201 published networks of interventions. BMC Med Res Methodol. 2021;21(1):224. 34689743 10.1186/s12874-021-01401-y PMC8543923

[13] Song F, Clark A, Bachmann MO, et al. Simulation evaluation of statistical properties of methods for indirect and mixed treatment comparisons. BMC Med Res Methodol. 2012;12:138. 22970794 10.1186/1471-2288-12-138 PMC3524036

[14] Altman DG, Bland JM. Absence of evidence is not evidence of absence. BMJ. 1995;311(7003):485. 7647644 10.1136/bmj.311.7003.485 PMC2550545

[15] Salanti G. Indirect and mixed-treatment comparison, network, or multiple-treatments meta-analysis: many names, many benefits, many concerns for the next generation evidence synthesis tool. Res Synth Methods. 2012;3(2):80–97. 26062083 10.1002/jrsm.1037

[16] Veroniki AA, Mavridis D, Higgins JP, Salanti G. Characteristics of a loop of evidence that affect detection and estimation of inconsistency: a simulation study. BMC Med Res Methodol. 2014;14:106. 25239546 10.1186/1471-2288-14-106 PMC4190337

[17] Spineli LM. Local inconsistency detection using the Kullback-leibler divergence measure. Syst Rev. 2024;13(1):261. 39420381 10.1186/s13643-024-02680-4 PMC11487772

[18] Kullback S, Leibler RA. On information and sufficiency. Ann Math Statist. 1951;22(1):79–86.

[19] Song F, Altman DG, Glenny AM, Deeks JJ. Validity of indirect comparison for estimating efficacy of competing interventions: empirical evidence from published meta-analyses. BMJ. 2003;326(7387):472. 12609941 10.1136/bmj.326.7387.472 PMC150178

[20] Song F, Harvey I, Lilford R. Adjusted indirect comparison may be less biased than direct comparison for evaluating new pharmaceutical interventions. J Clin Epidemiol. 2008;61(5):455–63. 18394538 10.1016/j.jclinepi.2007.06.006

[21] Song F, Xiong T, Parekh-Bhurke S, Loke YK, Sutton AJ, Eastwood AJ, et al. Inconsistency between direct and indirect comparisons of competing interventions: meta-epidemiological study. BMJ. 2011;343:d4909. 21846695 10.1136/bmj.d4909 PMC3156578

[22] Papakonstantinou T. nmadb: Network Meta-Analysis Database API. R package version 1.2.0; 2019. https://CRAN.R-project.org/package=nmadb.

[23] Petropoulou M, Nikolakopoulou A, Veroniki AA, Rios P, Vafaei A, Zarin W, et al. Bibliographic study showed improving statistical methodology of network meta-analyses published between 1999 and 2015. J Clin Epidemiol. 2017;82:20–8. 27864068 10.1016/j.jclinepi.2016.11.002

[24] Mansournia MA, Geroldinger A, Greenland S, Heinze G. Separation in logistic regression: causes, consequences, and control. Am J Epidemiol. 2018;187(4):864–70. 29020135 10.1093/aje/kwx299

[25] van Valkenhoef G, Dias S, Ades AE, Welton NJ. Automated generation of node-splitting models for assessment of inconsistency in network meta-analysis. Res Synth Methods. 2016;7(1):80–93. 26461181 10.1002/jrsm.1167 PMC5057346

[26] Lu G, Ades AE. Assessing evidence inconsistency in mixed treatment comparisons. J Am Stat Assoc. 2006;101(474):447–59.

[27] Van Valkenhoef G, Kuiper J. gemtc: Network Meta-Analysis Using Bayesian Methods. R package Version 1.0–2, 2023. https://CRAN.R-project.org/package=gemtc.

[28] Spineli LM. rnmamod: Bayesian Network Meta-analysis with Missing Participants. R package version 0.5.0; 2025. https://CRAN.R-project.org/package=rnmamod.

[29] Spiegelhalter DJ, Abrams KR, Myles J. Prior distributions. In: Spiegelhalter DJ, Abrams KR, Myles JP, editors. Bayesian approaches to clinical trials and health-care evaluation. Chichester, England: John Wiley & Sons; 2004. p. 139–80.

[30] R Core Team. R: A Language and Environment for Statistical Computing, version 4.5.1. R Foundation for Statistical Computing, Vienna, Austria; 2025.https://www.R-project.org/.

[31] Gelman A, Rubin DB. Inference from iterative simulation. Stat Sci. 1992;7:457–511.

[32] Turner RM, Jackson D, Wei Y, Thompson SG, Higgins JP. Predictive distributions for between-study heterogeneity and simple methods for their application in Bayesian meta-analysis. Stat Med. 2015;34(6):984–98. 25475839 10.1002/sim.6381 PMC4383649

[33] Bakbergenuly I, Hoaglin DC, Kulinskaya E. Pitfalls of using the risk ratio in meta-analysis. Res Synth Methods. 2019;10(3):398–419. 30854785 10.1002/jrsm.1347 PMC6767076

[34] Wickham H. ggplot2: elegant graphics for data analysis. New York: Springer-Verlag; 2016.

[35] Kassambara A. ggpubr: "ggplot2" Based Publication Ready Plots. R package version 0.6.0; 2023. https://CRAN.R-project.org/package=ggpubr.

[36] Kiefer C, Sturtz S, Bender R. A simulation study to compare different estimation approaches for network meta-analysis and corresponding methods to evaluate the consistency assumption. BMC Med Res Methodol. 2020;20(1):36. 32093605 10.1186/s12874-020-0917-3 PMC7041240

[37] Qin L, Zhao S, Guo W, Tong T, Yang K. A comparison of two models for detecting inconsistency in network meta-analysis. Res Synth Methods. 2024;15(6):851–71. 38965066 10.1002/jrsm.1734

[38] Nikolakopoulou A, Higgins JPT, Papakonstantinou T, Chaimani A, Del Giovane C, Egger M, et al. CINeMA: an approach for assessing confidence in the results of a network meta-analysis. PLoS Med. 2020;17(4):e1003082. 32243458 10.1371/journal.pmed.1003082 PMC7122720

