# Supplementary material for: Inconsistency was more prevalent than reported: an empirical study of 57 networks with multiple treatments using the node-splitting approach and a novel interpretation index
Source: Syst Rev. 2025 Nov 28;14:240. doi: 10.1186/s13643-025-02984-z (PMC12661744; doi:10.1186/s13643-025-02984-z)
Supplement: Supplementary file 1 — Additional file 1: Methods A. Local inconsistency evaluation using the node-splitting approach. Methods B. Parabola-like association of the index ${D}^{j}$Dj with inconsistency. Method C. Selecting predictive distributions for the between-study variance. [file 13643_2025_2984_MOESM1_ESM.docx]

**Additional file 1**

**Supplementary material for the manuscript entitled 'Inconsistency was more prevalent than reported: an empirical study of 57 networks with multiple treatments using the node-splitting approach and a novel interpretation index'**

Loukia M. Spineli^1^  [Spineli.Loukia@mh-hannover.de](mailto:Spineli.Loukia@mh-hannover.de)

^1^Midwifery Research and Education Unit, Hannover Medical School, Hannover, Germany

**Methods A: Local inconsistency evaluation using the node-splitting approach**

The node-splitting approach is a generalisation of the loop-specific method [8] for more complex network structures. Dias et al. [9] illustrated the node-splitting approach using a hierarchical Bayesian framework. In short, a comparison (node) found in a closed loop of evidence is initially isolated (split) from the network [9]. Then, a random-effects network meta-analysis (NMA) and pairwise meta-analysis (PMA) are applied in the remaining network and the split node, respectively, yielding a posterior distribution for the indirect estimate of that node based on NMA and a posterior distribution for the corresponding direct estimate through PMA [9]. The difference between the direct and indirect estimates provides an estimate of inconsistency (posterior distribution) for the split node [9]. A corresponding Bayesian two-sided p-value can be estimated [9]. The between-study variance parameter ($\tau^{2}$) is estimated using all available evidence by allowing the parameter to be shared between NMA and PMA [9].

The node-splitting approach was further refined by van Valkenhoef et al. [25] to facilitate the automatic splitting of necessary nodes when multi-arm studies are present and multiple loops shape the network structure. The candidate node to split should belong to a closed loop of at least three comparisons, with none of the comparisons sharing the same set of studies [25,26]. The gemtc R package [27] automatically detects the nodes to split through the *mtc.nodesplit.comparisons* function. This function has been integrated into the rnmamod package [28] to run the node-splitting approach.

**Methods B: Parabola-like association of the index** $\boldsymbol{D}^{\boldsymbol{j}}$ **with inconsistency**

The interpretation index $D^{j}$ for a split node $j$ is the average of $D_{D,I}^{j}$ (the KLD for approximating the distribution of the direct estimate with that of the corresponding indirect estimate) and $D_{I,D}^{j}$ (the KLD for approximating the distribution of the indirect estimate with that of the corresponding direct estimate):

$$D^{j}=\frac{D_{D,I}^{j}+D_{I,D}^{j}}{2}$$

$$=\frac{1}{2}\left\{ \frac{1}{2}\left[ \frac{\hat{s}_{D}^{2}}{\hat{s}_{I}^{2}}+\frac{\left( \hat{\mu}_{D}-\hat{\mu}_{I} \right)^{2}}{\hat{s}_{I}^{2}}-1+ln\left( \frac{\hat{s}_{I}^{2}}{\hat{s}_{D}^{2}} \right) \right]+\frac{1}{2}\left[ \frac{\hat{s}_{I}^{2}}{\hat{s}_{D}^{2}}+\frac{\left( \hat{\mu}_{D}-\hat{\mu}_{I} \right)^{2}}{\hat{s}_{D}^{2}}-1+ln\left( \frac{\hat{s}_{D}^{2}}{\hat{s}_{I}^{2}} \right) \right] \right\}$$

$$=\frac{1}{4}\left[ \frac{\hat{s}_{D}^{2}}{\hat{s}_{I}^{2}}+\frac{\left( \hat{\mu}_{D}-\hat{\mu}_{I} \right)^{2}}{\hat{s}_{I}^{2}}-1+ln\left( \frac{\hat{s}_{I}^{2}}{\hat{s}_{D}^{2}} \right)+\frac{\hat{s}_{I}^{2}}{\hat{s}_{D}^{2}}+\frac{\left( \hat{\mu}_{D}-\hat{\mu}_{I} \right)^{2}}{\hat{s}_{D}^{2}}-1+ln\left( \frac{\hat{s}_{D}^{2}}{\hat{s}_{I}^{2}} \right) \right]$$

$$=\frac{\hat{s}_{D}^{4}+\hat{s}_{I}^{4}-2\hat{s}_{D}^{2}\hat{s}_{I}^{2}}{4\hat{s}_{D}^{2}\hat{s}_{I}^{2}}+\left( \hat{\mu}_{D}-\hat{\mu}_{I} \right)^{2}\frac{\hat{s}_{D}^{2}+\hat{s}_{I}^{2}}{4\hat{s}_{D}^{2}\hat{s}_{I}^{2}}$$

$$=\frac{\left( \hat{s}_{D}^{2}-\hat{s}_{I}^{2} \right)^{2}}{4\hat{s}_{D}^{2}\hat{s}_{I}^{2}}+\left( \hat{\mu}_{D}-\hat{\mu}_{I} \right)^{2}\frac{\hat{s}_{D}^{2}+\hat{s}_{I}^{2}}{4\hat{s}_{D}^{2}\hat{s}_{I}^{2}}.$$

Therefore, it holds that:

$$D^{j}=ax^{2}+bx+c$$

with

$$a=\frac{\hat{s}_{D}^{2}+\hat{s}_{I}^{2}}{4\hat{s}_{D}^{2}\hat{s}_{I}^{2}}\text{,} b=0\text{,} c=\frac{\left( \hat{s}_{D}^{2}-\hat{s}_{I}^{2} \right)^{2}}{4\hat{s}_{D}^{2}\hat{s}_{I}^{2}} \text{and} x=\hat{\mu}_{D}-\hat{\mu}_{I}.$$

Parameter $a$ controls the shape of the parabola, with the parabola becoming wider upwards for increasing values of $a$ ($a>0$). Parameter $c$ causes the parabola to move only upwards because $c\geq0$.

**Methods C: Selecting predictive distributions for the between-study variance**

There have been proposed predictive distributions for the between-study variance, $\tau^{2}$, based on several meta-analyses of odds ratios in the logarithmic scale tailored to the outcome type (i.e., objective, semi-objective, and subjective) and treatment-comparison type (i.e., pharmacological versus placebo, pharmacological versus pharmacological, and non-pharmacological versus any) that can be used as informative priors for $\tau^{2}$ in the analysis [32]. We adopted the designations found in the nmadb R package [22] for each eligible network about the outcome and treatment-comparison types.

Since Turner et al. [32] considered several semi-objective and subjective outcomes (see Table II in [32]), we applied the predictive distribution with the largest median value for each outcome type, which is a conservative suggestion. Table S1 summarises the predictive distributions per outcome and treatment-comparison types considered in the present study.

**Table S1.** Predictive distributions for $\tau^{2}$ considered in the present study

| **Outcome type** | **Treatment-comparison type** | | |
| --- | --- | --- | --- |
|  | **pharma *vs*. placebo** | **pharma *vs*. pharma** | **non-pharma *vs*. pharma** |
| *Binary outcomes* | | | |
| Objective | $LN(-3.95, {1.34}^{2})$ | $LN(-4.18, {1.41}^{2})$ | $LN(-2.92, {1.02}^{2})$ |
| Semi-objective | $LN(-2.14, {1.74}^{2})$ | $LN(-2.37, {1.79}^{2})$ | $LN(-1.11, {1.50}^{2})$ |
| Subjective | $LN(-1.77, {1.52}^{2})$ | $LN(-2.00, {1.58}^{2})$ | $LN(-0.74, {1.24}^{2})$ |

LN, log-normal distribution.
