# Supplementary material for: Inconsistency was more prevalent than reported: an empirical study of 57 networks with multiple treatments using the node-splitting approach and a novel interpretation index
Source: Syst Rev. 2025 Nov 28;14:240. doi: 10.1186/s13643-025-02984-z (PMC12661744; doi:10.1186/s13643-025-02984-z)
Supplement: Supplementary file 2 — Additional file 2: Figure S1. An illustrative example of the interpretation index ${D}^{j}$Dj under an acceptably low and material inconsistency. Figure S2. Scatter plots of the posterior standard deviation against the estimated a) direct effects, b) indirect effects, c) inconsistency factor, and d) between-study standard deviation of 404 split nodes in 57 networks. Figure S3. Stacked bar plots on the conclusions about inconsistency based on the interpretation index ${D}^{j}$Dj and the 95% credible interval of inconsistency factor. Figure S4. Violin plots on the distribution of the interpretation index ${D}^{j}$Dj among split nodes with one and more studies. Figure S5. Scatter plot of the posterior standard deviation of inconsistency against the posterior mean of inconsistency factor for split nodes with a) material inconsistency, and b) acceptably low inconsistency. Figure S6. Scatter plot of the interpretation index ${D}^{j}$Dj against [file 13643_2025_2984_MOESM2_ESM.docx]

**Additional file 2**

**Supplementary material for the manuscript entitled 'Inconsistency was more prevalent than reported: an empirical study of 57 networks with multiple treatments using the node-splitting approach and a novel interpretation index'**

Loukia M. Spineli^1^  [Spineli.Loukia@mh-hannover.de](mailto:Spineli.Loukia@mh-hannover.de)

^1^Midwifery Research and Education Unit, Hannover Medical School, Hannover, Germany


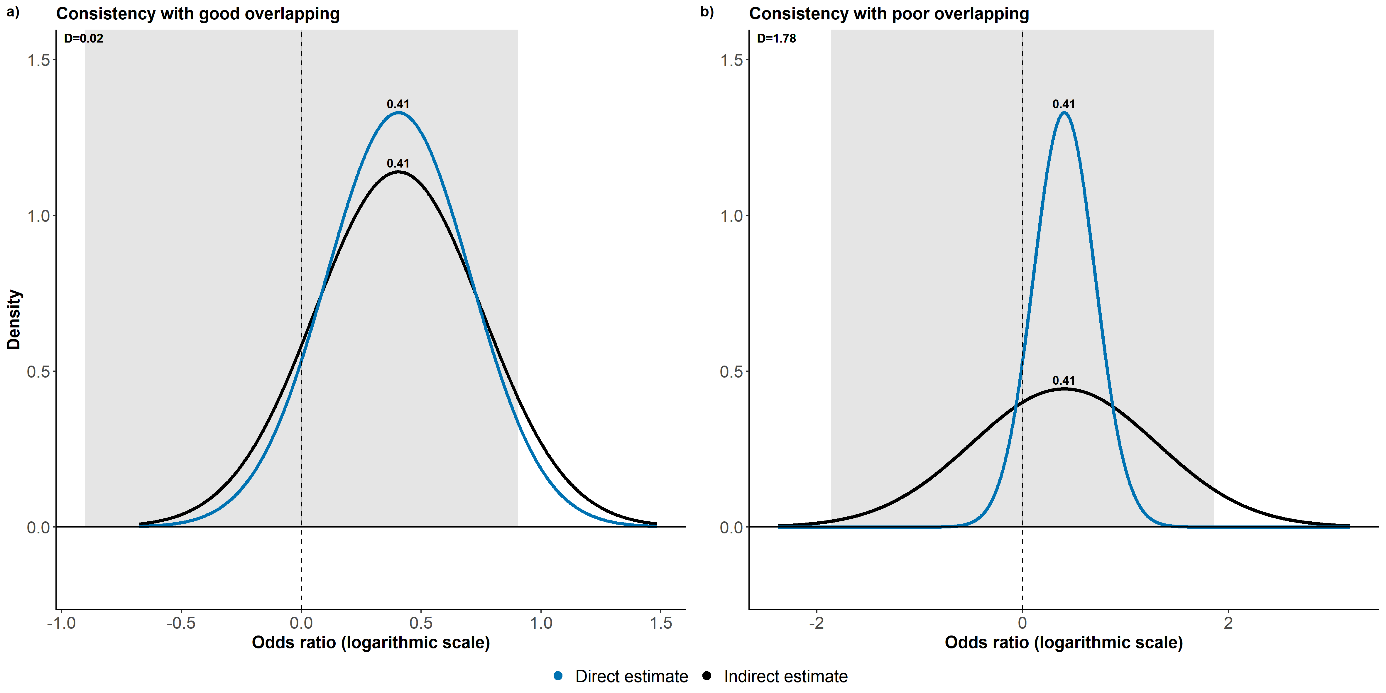


**Figure S1.** Probability densities of the direct (blue line) and indirect (black line) odds ratio in the logarithmic scale (log OR) for a fictional split node assuming $\hat{\mu}_{D}=\hat{\mu}_{I}=ln(1.5)$ (consistency on average). In both plots, $\hat{s}_{D}^{2}={0.30}^{2}$ for the direct log OR. For the indirect log OR, $\hat{s}_{I}^{2}={0.35}^{2}$ in plot a), resulting in $D^{j}$ equal to 0.02 (good distribution overlap), and $\hat{s}_{I}^{2}={0.90}^{2}$ in plot b), yielding $D^{j}$ equal to 1.78 (poor distribution overlap). The grey vertical line and grey area indicate the superimposed inconsistency factor and 95% interval *approximated* using either estimate's reported mean and variance; namely, $\left( \hat{\mu}_{D}-\hat{\mu}_{I} \right)\pm1.96\sqrt{\hat{s}_{D}^{2}+\hat{s}_{I}^{2}}$.

**Interpreting the Figure:** In both scenarios, the 95% interval for the inconsistency factor includes the zero value, signalling inconclusive inconsistency (in the frequentist framework, it would coincide with a p-value above the selected significance threshold). The indirect log OR has a comparatively larger variance than the direct log OR, especially in Figure 1b). In Figure 1a) both distributions overlap substantially, yielding an almost zero $D^{j}$. Assuming a larger variance for the indirect log OR ($\hat{s}_{I}^{2}={0.90}^{2}$) in Figure 1b) led to an insufficient overlap of the probability distributions for the split node, yielding a large $D^{j}$ at 1.78, which exceeded the threshold of 0.64, signaling material inconsistency.


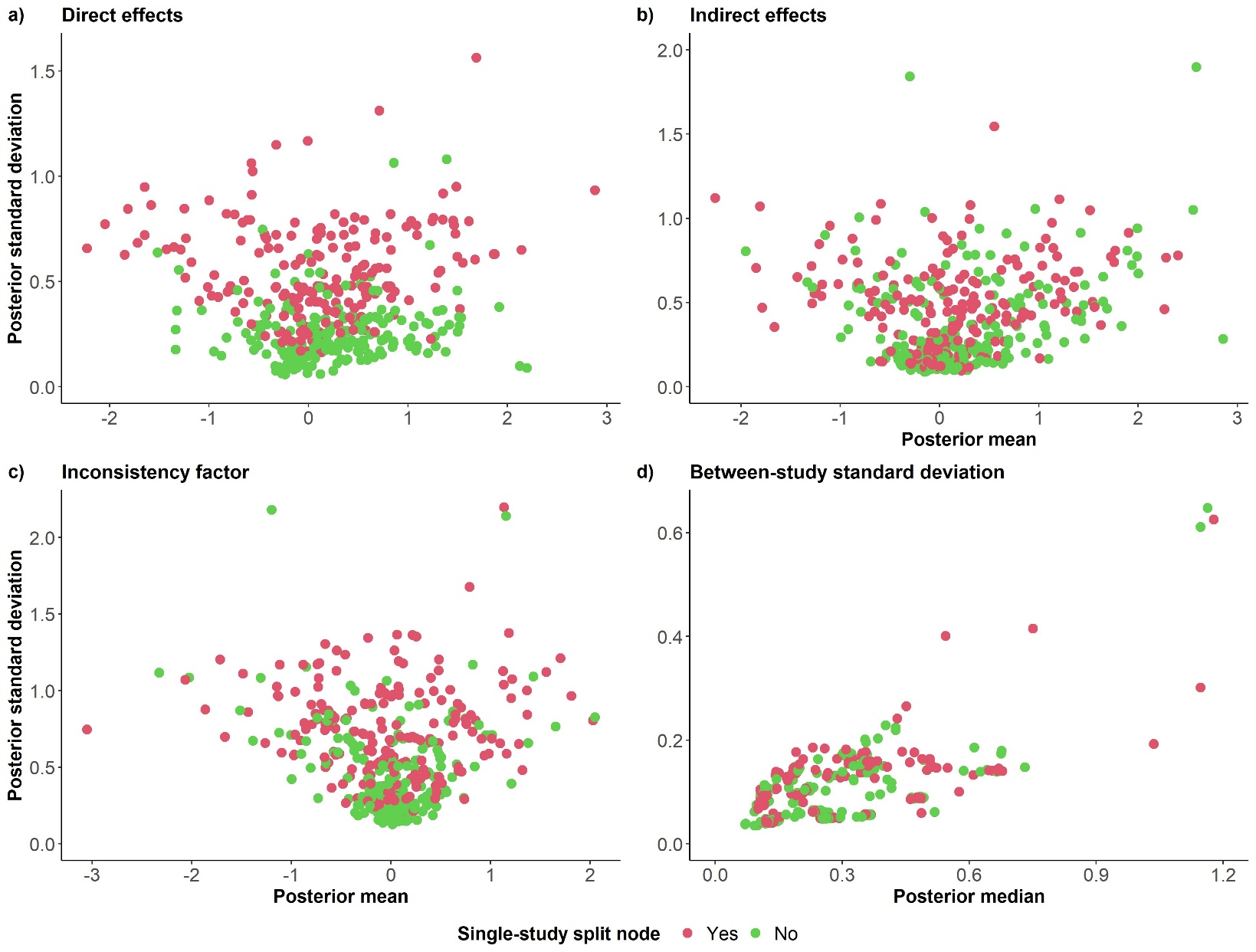


**Figure S2.** Scatter plots of the posterior standard deviation against the posterior mean or median of a) direct effects, b) indirect effects, c) inconsistency factor, and d) between-study standard deviation of 404 split nodes in 57 networks. Red and green dots refer to split nodes with one study and more studies.


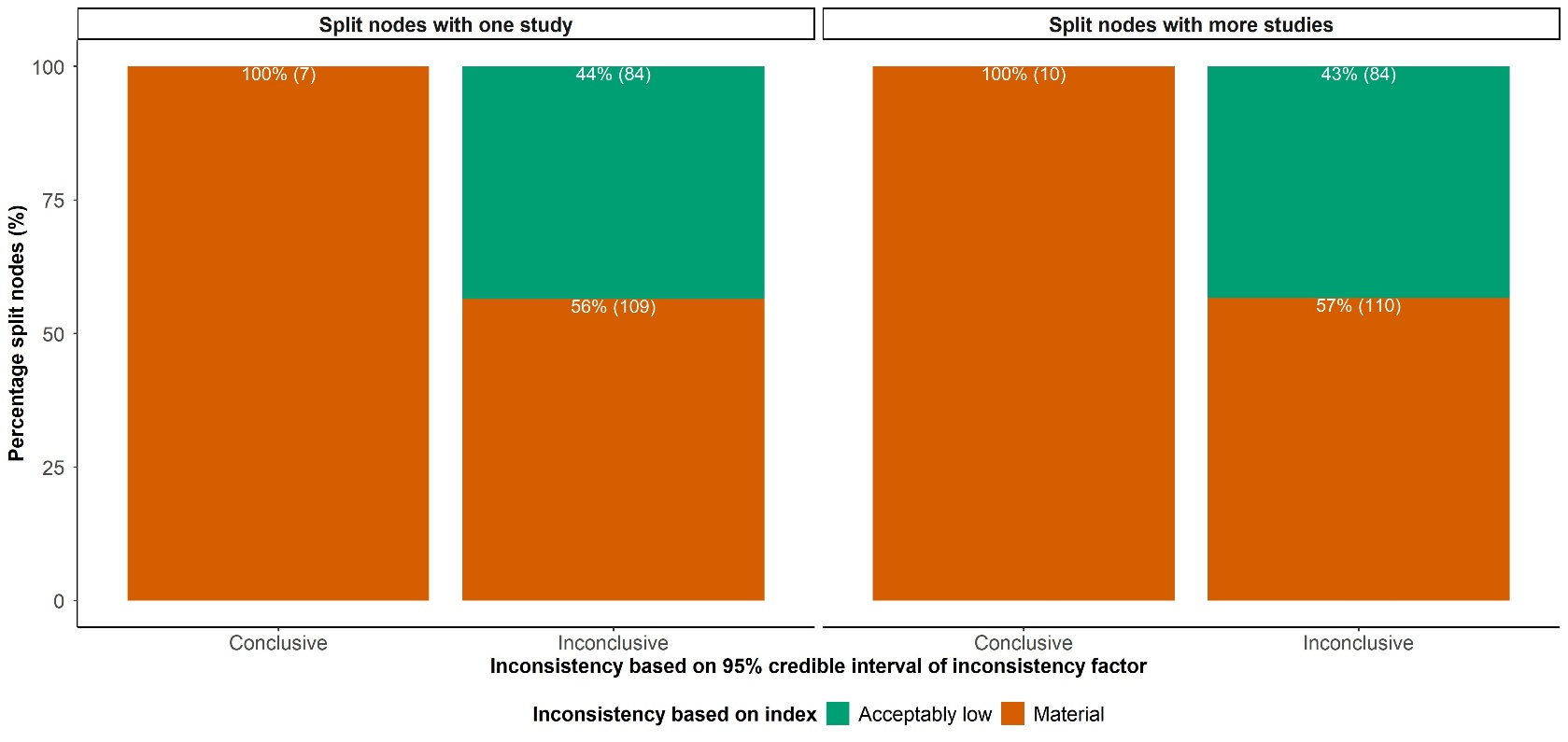


**Figure S3.** Stacked bar plots with the conclusions about inconsistency based on the interpretation index $D^{j}$ and the 95% credible interval of the inconsistency factor in 200 single-study split nodes and 204 split nodes with more studies. Percentages refer to split nodes with acceptably low ($D^{j}<0.64$) or material inconsistency based on the index $D^{j}$ out of those with conclusive (95% credible interval excludes zero inconsistency) and inconclusive inconsistency.


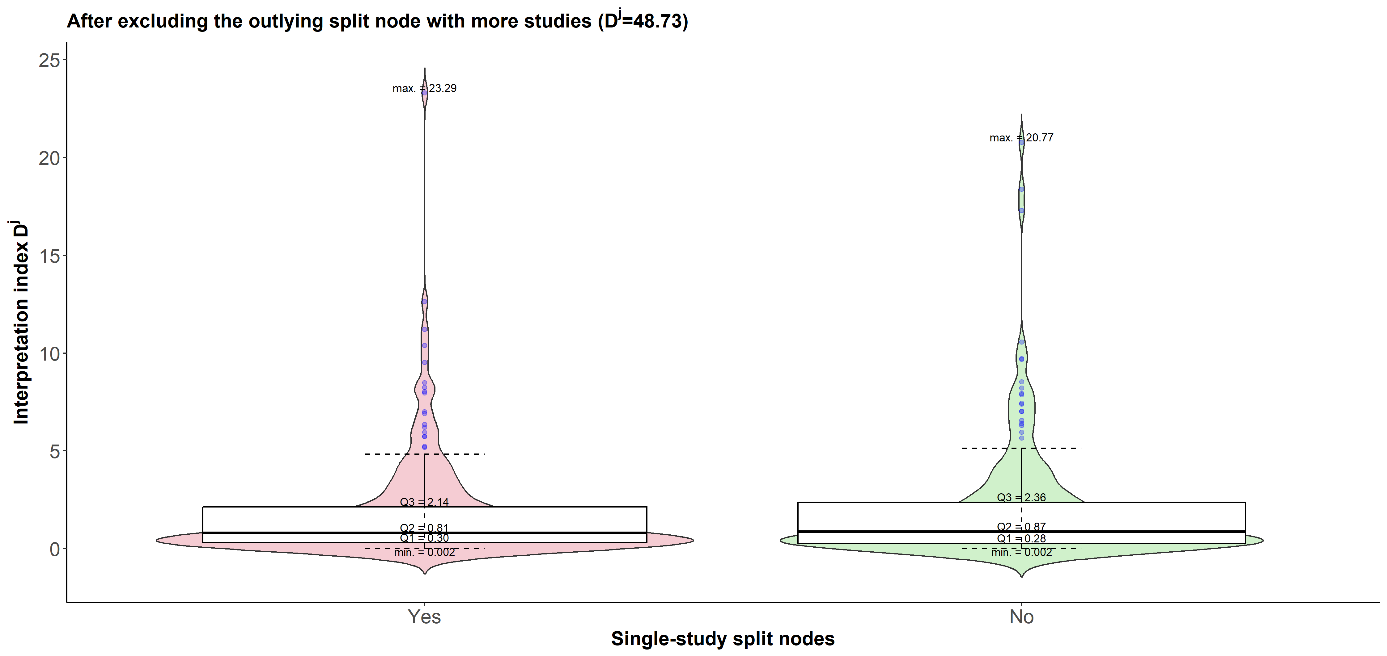


**Figure S4.** Violin plots with integrated box plots and dots on the distribution of the interpretation index $D^{j}$ among 200 single-study split nodes (red violin) and 204 split nodes with more studies (green violin). The five quartiles appear on each violin plot. min., minimum; max., maximum; Q1, first quartile; Q2, second quartile (the median); Q3, third quartile.

**
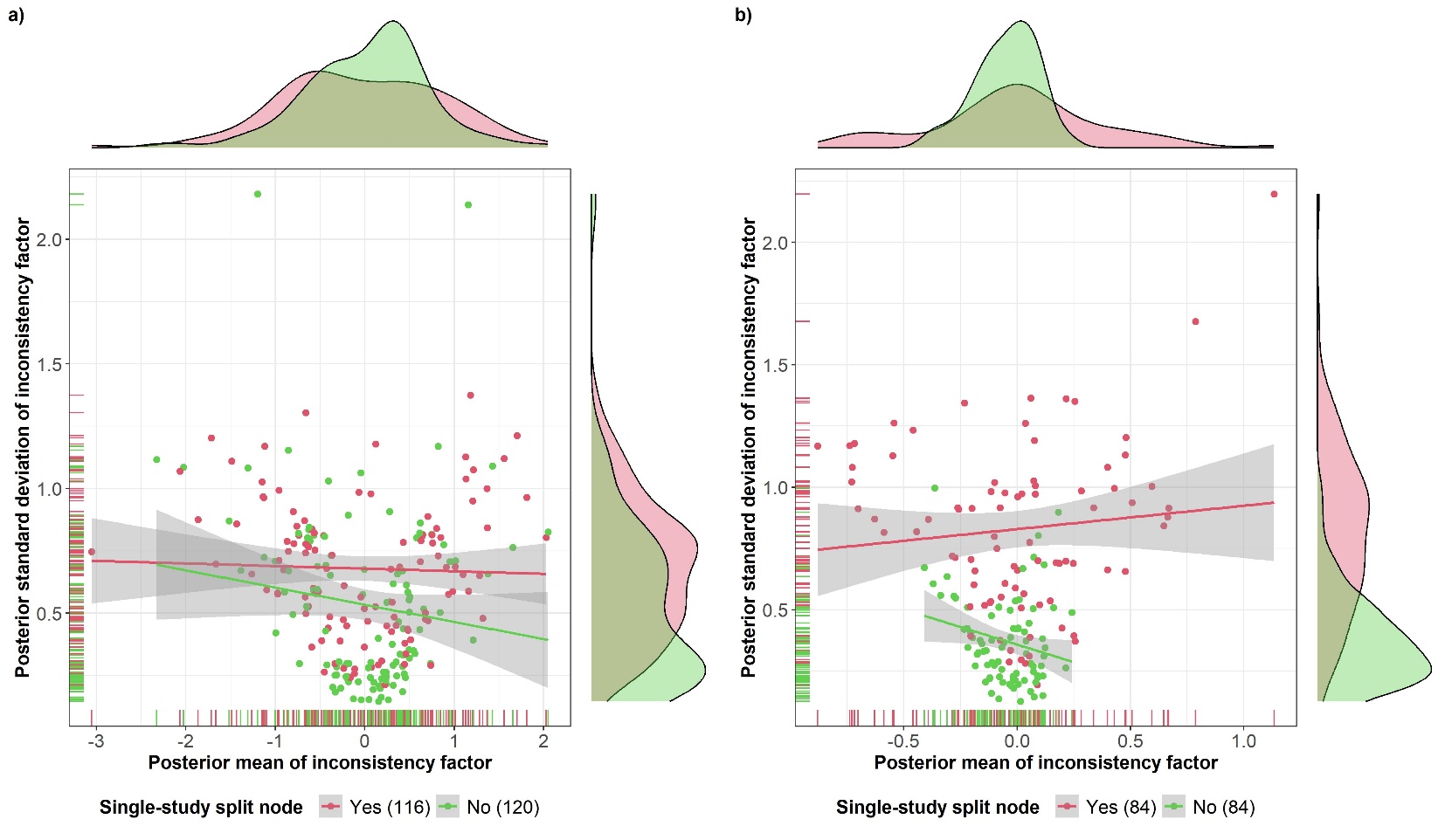
**

**Figure S5.** Scatter plot of the posterior standard deviation of the inconsistency factor against the posterior mean of the inconsistency factor for split nodes with a) material inconsistency ($D^{j}\geq0.64$), and b) acceptably low inconsistency. Red and green refer to split nodes with one study and more studies. A density plot of the distribution of the posterior mean of the inconsistency factor for each split node group is appended above the scatter plots, and a density plot of the distribution of the posterior standard deviation of the inconsistency factor for each split node group is appended on the right of the scatter plots.


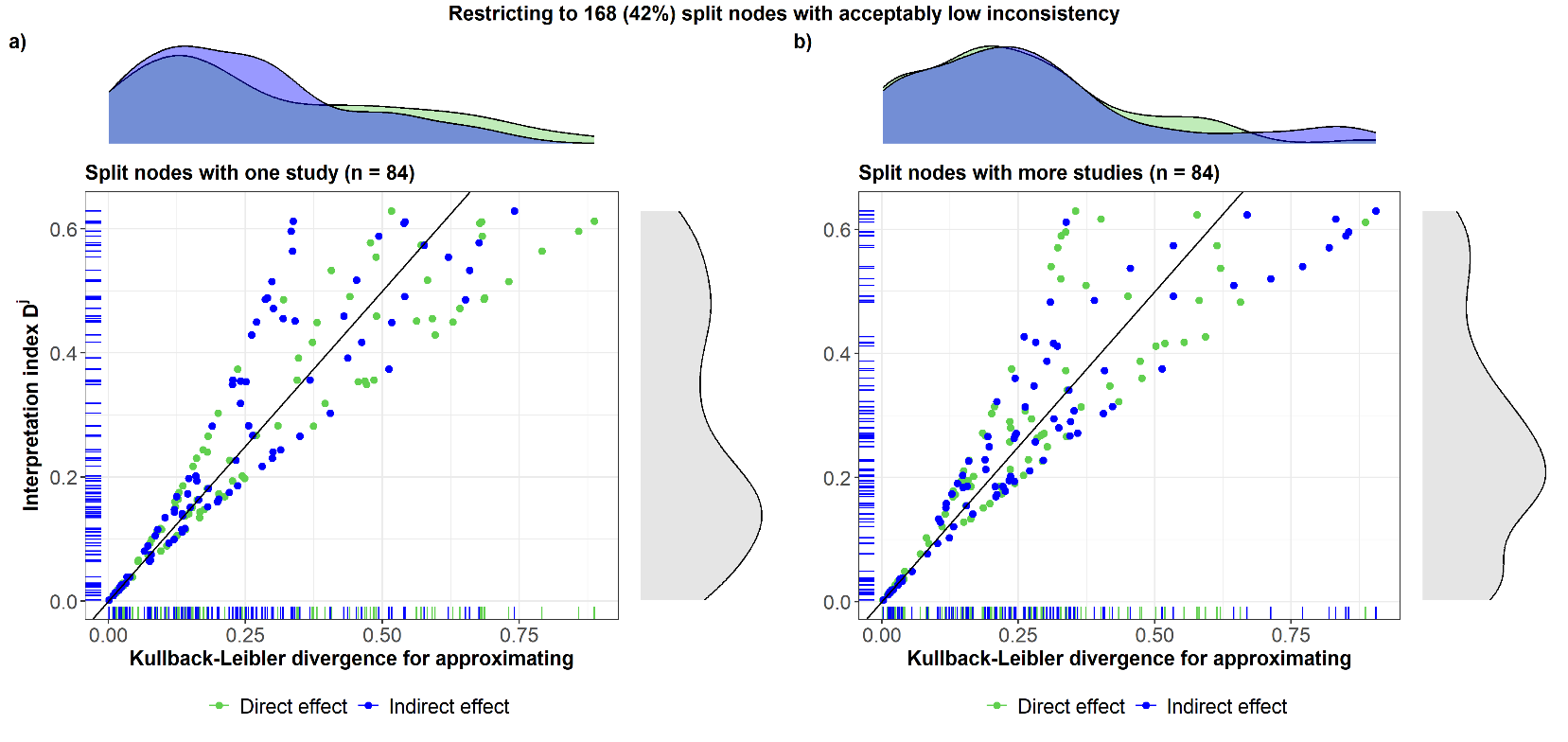


**Figure S6.** Scatter plot of the interpretation index $D^{j}$ against the Kullback-Leibler divergence of approximating the direct with the indirect effects ($D_{D,I}^{j}$, green dots) and Kullback-Leibler divergence of approximating the indirect with the direct effects ($D_{I,D}^{j}$, blue dots) in a) 84 single-study split nodes and b) 84 split nodes with more studies with acceptably low inconsistency ($D^{j}<0.64$). Both scatter plots have a black diagonal line. A density plot of the distribution of $D_{D,I}^{j}$ and $D_{I,D}^{j}$ is appended above the scatter plots and a density plot of the distribution of $D^{j}$ is appended on the right of the scatter plots.


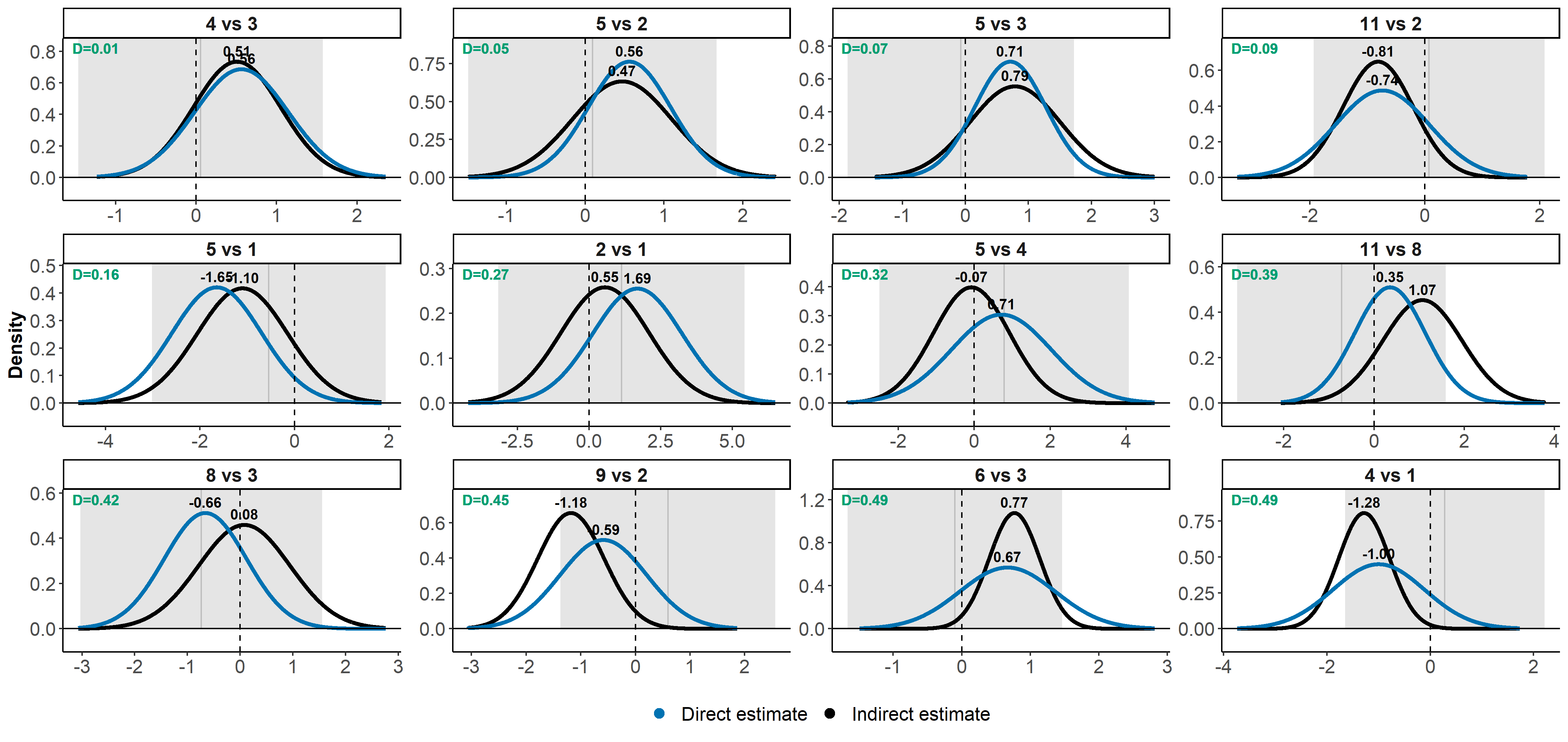


**Figure S7.** Posterior densities of the direct (blue line) and indirect (black line) log odds ratios for 12 split nodes with $D^{j}<0.64$ and fairly high or extreme statistical heterogeneity ($\tau\geq0.5$). The grey area and vertical line refer to the 95% interval and posterior mean of the inconsistency factor. The 95% interval has been approximated using the direct and indirect effects' posterior mean and standard deviation. The interpretation index $D^{j}$ appears at the top left of each plot. The plots have been sorted in ascending order of the $D^{j}$ values. The *x*-axis and *y*-axis values vary across all plots.


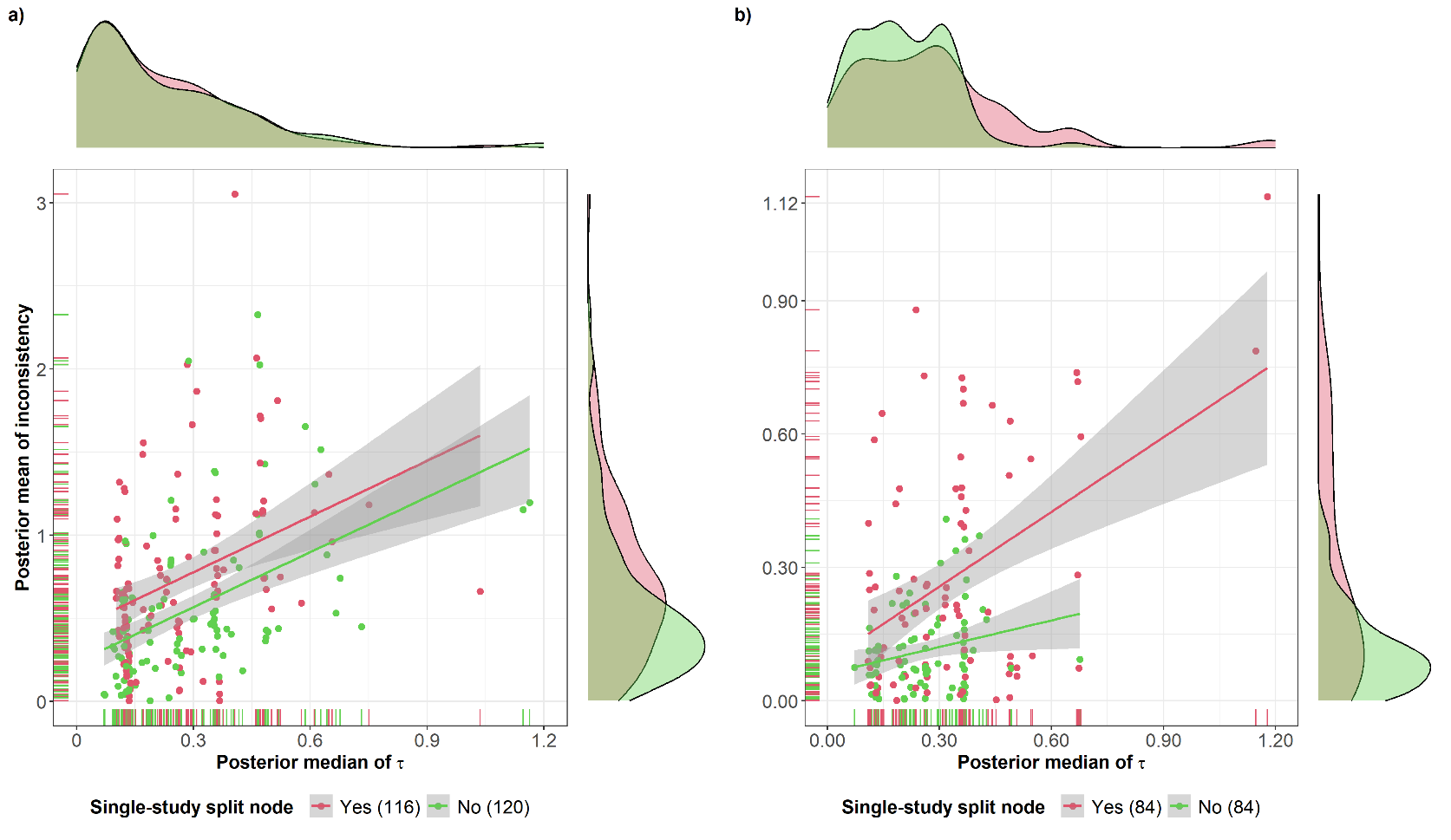


**Figure S8.** Scatter plot of the posterior mean of the inconsistency factor against the posterior median of $\tau$ for split nodes with a) material inconsistency ($D^{j}\geq0.64$), and b) acceptably low inconsistency. Red and green refer to split nodes with one study and more studies. A density plot of the distribution of $\tau$ for each split node group is appended above the scatter plots, and a density plot of the distribution of the posterior mean of the inconsistency factor for each split node group is appended on the right of the scatter plots.
